# Roles of microRNAs in mammalian reproduction: from the commitment of germ cells to peri‐implantation embryos

**DOI:** 10.1111/brv.12459

**Published:** 2018-08-27

**Authors:** Abu Musa Md Talimur Reza, Yun‐Jung Choi, Sung Gu Han, Hyuk Song, Chankyu Park, Kwonho Hong, Jin‐Hoi Kim

**Affiliations:** ^1^ Department of Stem Cell and Regenerative Biotechnology Humanized Pig Research Centre (SRC), Konkuk University Seoul 143‐701 Republic of Korea; ^2^ Department of Food Science and Biotechnology of Animal Resources Konkuk University Seoul 05029 Republic of Korea

**Keywords:** miRNAs, mammalian reproduction, gametogenesis, hormonal balance, peri‐implantation, germ layer specification

## Abstract

MicroRNAs (miRNAs) are active regulators of numerous biological and physiological processes including most of the events of mammalian reproduction. Understanding the biological functions of miRNAs in the context of mammalian reproduction will allow a better and comparative understanding of fertility and sterility in male and female mammals. Herein, we summarize recent progress in miRNA‐mediated regulation of mammalian reproduction and highlight the significance of miRNAs in different aspects of mammalian reproduction including the biogenesis of germ cells, the functionality of reproductive organs, and the development of early embryos. Furthermore, we focus on the gene expression regulatory feedback loops involving hormones and miRNA expression to increase our understanding of germ cell commitment and the functioning of reproductive organs. Finally, we discuss the influence of miRNAs on male and female reproductive failure, and provide perspectives for future studies on this topic.

## I. INTRODUCTION

Mammalian microRNAs (miRNAs) are non‐coding RNAs that can regulate post‐transcriptional gene expression by targeting the 3′‐untranslated region and/or coding region of messenger RNAs (mRNAs) (Hausser *et al.,* 2013), and are involved in the regulation of approximately one‐third of all mammalian genes (Lewis, Burge, & Bartel, 2005). While a single miRNA may target many genes, the expression of a particular gene can be modulated by multiple miRNAs (Sood *et al.,* 2006). Hence, miRNAs are involved in the active regulation of biological and physiological processes in both normal and disease conditions (Reza *et al.,* 2016, 2017, 2018a; Reza, Choi, & Kim, 2018b). In mammalian reproduction, miRNAs are widely involved in sex differentiation (Cook & Blelloch, 2013), gametogenesis (Hayashi *et al.,* 2008), fertilization (Hong *et al.,* 2008), zygotic genome activation (ZGA) and early development (Suh *et al.,* 2010), implantation (Liu *et al.,* 2016), germ layer specification (Vidigal & Ventura, 2012), and pregnancy (Otsuka *et al.,* 2008). Understanding the functions of miRNAs in mammalian reproduction will therefore improve our understanding of the biological challenges of mammalian reproduction.

Several reviews have focused on the event‐specific involvement of non‐coding RNAs (miRNAs, endogenous siRNAs, piwi‐interacting RNAs) in the reproduction of different species. However, those reviews included all types of non‐coding RNAs and a wide range of species (insects, non‐vertebrates, fishes, and birds), in order to develop an understanding of their roles in reproductive biology. To comprehend specifically the roles of miRNAs in mammalian reproduction requires an in‐depth review focusing on mammalian species, as mammals are relatively unique in their structure, physiology, and psychology. Furthermore, the available reviews mainly focused on particular aspects of reproduction such as oogenesis (Donadeu, Schauer, & Sontakke, 2012; Hossain *et al.,* 2012a), spermatogenesis (Kotaja, 2014; Yadav & Kotaja, 2014; Hilz *et al.,* 2016; Chen *et al.,* 2017), early embryonic development (Suh & Blelloch, 2011; Cook & Blelloch, 2013; Dallaire & Simard, 2016), or implantation (Liu & Yang, 2011; Dior *et al.,* 2014; Galliano & Pellicer, 2014; Liu *et al.,* 2016), with a holistic review focusing on all processes of mammalian reproduction lacking. Such a holistic review focusing on miRNA involvement in all aspects of mammalian reproduction is necessary to improve our understanding of reproductive biology and to mitigate mammalian reproductive challenges.

Herein, we summarize recent progress in miRNA‐mediated regulation of mammalian reproduction, and highlight the significance of miRNAs in different aspects of mammalian reproduction including the biogenesis of germ cells, functionality of reproductive organs, and development of early embryos, as well as covering infertility and sterility derived from miRNA deregulation in males and females. First, we discuss impairments and abnormalities caused by depletion of miRNA‐processing machinery during germ cell commitment and the functioning of reproductive organs. Second, we discuss how miRNAs interfere at different stages of male and female reproduction. Furthermore, we focus on the distribution and dynamics of miRNAs during gametogenesis, fertilization, embryo development, and implantation, as well as specification to three different germ layers. Finally, we discuss the influence of miRNAs on male and female reproductive failure, and provide a set of conclusive remarks to guide future studies in this topic.

## II. LOSS OF miRNA‐PROCESSING MACHINERY IMPAIRS MAMMALIAN REPRODUCTION

**Figure 1:**
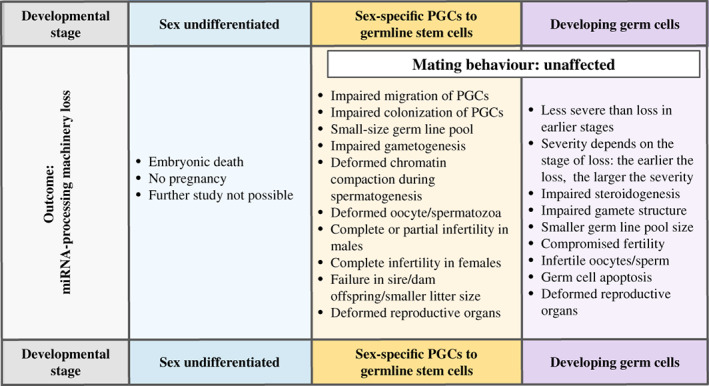
Adverse effects of the loss of microRNA (miRNA)‐processing machinery during mammalian reproduction. The loss of miRNA‐processing machinery can cause reproductive impairment to different extents, including embryonic death, based on the stage at which it is lost. However, mating behaviour of both males and females remains intact. PGC, primordial germ cell.

Loss of one or both of the components of the miRNA‐processing machinery (Dicer and Drosha) causes severe impairment during gametogenesis, leading to infertility in both males and females (Fig. 1). Especially, Dicer and Drosha are indispensable during the early stages of development such as before sex differentiation (Bernstein *et al.,* 2003), while in the later stages, their loss causes impairment to different extents depending on the stage at which Dicer or Drosha is deleted (Hong *et al.,* 2008; Liu *et al.,* 2010; Liu *et al.,* 2012). However, it is clear that this impairment does not arise from the absence of the proteins themselves, but rather from the associated failure of biogenesis of small‐RNAs (miRNAs and endogenous siRNAs) (Papaioannou *et al.,* 2009; Hossain *et al.,* 2012b). The severity of *Dicer1* deletion is highly stage and organ specific, with other organs or processes often remaining functional. Interestingly, sexual behaviour, mounting ability, external genitalia, and oestrous are not affected by the depletion of Dicer (Papaioannou *et al.,* 2009).

In the male, primordial germ cell (PGC)‐specific deletion of Dicer and Drosha restricts the propagation, differentiation, and maturation of male PGCs (Hayashi *et al.,* 2008) and causes defective spermatozoa and infertility (Maatouk *et al.,* 2008). Spermatogonia‐specific ablation of *Dicer1* impairs the leptotene to zygotene/pachytene transition during prophase I with an increase in apoptotic pachytene spermatocytes, deformed chromatin compaction, acrosome formation, and sperm‐head structure (Romero *et al.,* 2011). Round spermatid‐specific deletion of *Dicer1* deregulated *actin related protein 2/3 complex subunit 5* (*Arpc5*), leading to abnormal chromatin compaction and causing deformed acrosomes and sperm heads, as well as apoptosis of spermatids in mice (Chang *et al.,* 2012). Sertoli cell‐specific deletion of *Dicer1* caused apoptosis of Sertoli cells after birth leading to degenerated testes, defective prepubertal spermatogenesis, and infertility (Papaioannou *et al.,* 2009). Epididymis‐specific deletion of *Dicer1* resulted in imbalanced lipid homeostasis and instability of the sperm membrane (Bjorkgren *et al.,* 2015) as well as leakage of immature spermatocytes to the epididymis and a thick blood–testis barrier with a multilayered organization (Korhonen *et al.,* 2015). Hence, Dicer/Drosha safeguard meiotic progression and the production of spermatozoa, as well as the development of accessory organs, by supplying functional miRNAs.

In females, *Dicer1* loss causes abnormal spindle formation and chromosomal misalignment (Liu *et al.,* 2010), failures in meiotic maturation and polar body formation (Murchison *et al.,* 2007; Liu *et al.,* 2010) and functional defects including impaired granulosa cell (GC) proliferation and steroidogenesis, recruitment of immature follicles, follicular atresia, a smaller pool size of pre‐ovulatory follicles, and ovulatory dysfunction followed by infertility (Murchison *et al.,* 2007; Hong *et al.,* 2008; Lei *et al.,* 2010). *Dicer1* deficiency impairs the biogenesis of small‐RNAs altering gene expression and signalling networks: *Dicer1*‐ablated ovaries show deregulation of miR‐503 and its targets including *anti‐mullerian hormone* (*Amh*), *inhibin beta A subunit* (*Inhba*), *cytochrome P450 family 17 subfamily A member 1* (*Cyp17a1*), *cytochrome P450 family 19 subfamily A member 1* (*Cyp19a1*), *zona pellucida glycoprotein* (*Zp*), *growth differentiation factor 9* (*Gdf9*), and *bone morphogenetic protein 15* (*Bmp15*), leading to impairment of folliculogenesis in mice (Lei *et al.,* 2010). Similarly, inhibition of *Dicer1* reduced the expression of *polo like kinase 1* (*Plk1*), *aurora kinase A* (*Aurka*), *BUB1 mitotic checkpoint serine/threonine kinase* (*Bub1*), and *BUB1 mitotic checkpoint serine/threonine kinase B* (*Bub1b*), enhanced abnormal spindle formation and chromosomal misalignment, and lowered the maturation rate of oocytes (Murchison *et al.,* 2007; Tang *et al.,* 2007; Liu *et al.,* 2010).

The replacement of *Dicer1*‐ablated ovaries with wild‐type (WT) ovaries enable the mice to produce viable offspring, while implantation of *Dicer1*‐ablated ovaries into WT mice results in failure to produce offspring (Otsuka *et al.,* 2008). The failure is prominent during post‐implantation development, while ovulation, fertilization, and first embryonic cell division appear normal (Otsuka *et al.,* 2008) and may be due to disintegration of the corpus luteum (CL) and lower progesterone levels (Otsuka *et al.,* 2008), which may derive from impairment to miRNA regulation required for the angiogenesis, maintenance, and functioning of the CL (Yang *et al.,* 2005; Otsuka *et al.,* 2008). Moreover, depletion of *Dicer1* causes disintegration of the structural, mechanical, and functional ability of the fallopian tube including loss of the smooth muscle layer, disorganized epithelium, and deregulation of signalling networks (Luense, Carletti & Christenson, 2009): this might also cause failures in the transportation and implantation of embryos to the uterus (Luense, Carletti, & Christenson, 2009), and the resulting offspring may have impaired reproductive organs because of maternal loss of oviductal *Dicer1* (Hong *et al.,* 2008). Fallopian tube‐specific loss of *Dicer1* results in shorter tubule length, abnormal coil formation and the presence of fluid‐filled sacs (Hong *et al.,* 2008; Gonzalez & Behringer, 2009). Taken together, the observations suggest that *Dicer1* in the female safeguards molecular and signalling networks related to gametogenesis, steroidogenesis, the genomic and structural architecture of the oocytes as well as the development and implantation of embryos.

## III. miRNAs ARE INVOLVED IN SEX‐SPECIFICATION AND COMMITMENT OF MAMMALIAN GONADS

**Figure 2:**
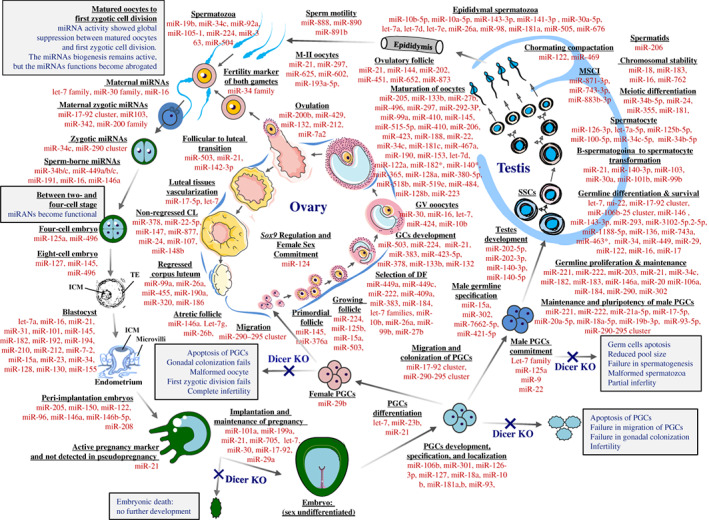
MicroRNA (miRNA) regulation at different stages of sex specification and gametogenesis. The miRNAs that are listed for different stages of oogenesis, spermatogenesis and early embryonic development are the predominantly expressed miRNAs of these stages. miRNAs are involved in every stage of early embryonic development and gametogenesis, except the period between the mature oocyte and first zygotic cell division, where the functions of miRNAs are abrogated (reported in mouse, but yet to be verified in other mammals), and then re‐established between two‐ and four‐cell stage pluripotent embryos. Loss of Dicer during the sex‐undifferentiated stage typically causes embryonic death, while primordial germ cell (PGC)‐specific loss causes the impairment and failure of migration, colonization, and commitment of sex‐specific gonads. miRNAs are highly expressed during gametogenesis, and some miRNAs have sex‐ and purpose‐specific roles; for example, miR‐124 plays an important role in female gonad commitment by suppressing *Sox‐9* expression, and the loss of miR‐124 before sex commitment can cause sex reversal in females. Other miRNAs are equally important in nearly all stages; for example, miR‐21 is important in all stages of gametogenesis and is responsible for the viability and survival of cells. The expression of miR‐21 during pregnancy indicates the presence of activated and live embryo (s). CL, corpus luteum; TE, trophectoderm; KO, knockout; GV, germinal vesicle; GC, granulosa cell; DF, dominant follicle; MSCI, meiotic sex chromosome inactivation.

Prior to sex determination PGCs carry a unique miRNA profile (Hayashi *et al.,* 2008) that does not match sex‐specific PGCs (Fig. 2), and PGCs undergoing sex determination display dimorphic expression patterns of miRNAs (see online Supporting information, Table S1). For example, the let‐7 family, miR‐125a, and miR‐9 are overexpressed in PGCs for male germline commitment (Hayashi *et al.,* 2008). By contrast, female germline‐committing PGCs show overexpression of miR‐29b, which targets *DNA methyltransferase 3 alpha* (*Dnmt3a*) and *DNA methyltransferase 3 beta* (*Dnmt3b*), and epigenetically regulates gene expression during oogenesis in mice (Takada *et al.,* 2009). Embryonic loss of miRNAs resulting from *Dicer1* deletion is associated with embryonic lethality in mice near the time of PGC specification (Bernstein *et al.,* 2003). *Dicer1* depletion just before PGC migration impaired the proliferation and gonad‐specific colonization of PGCs in mice (Hayashi *et al.,* 2008), and oocytes originating from *Dicer1*‐ablated mouse germline cells failed to establish functional zygotes and commence first zygotic cell division (Tang *et al.,* 2007). These findings indicate that sex differentiation and embryonic development require parentally derived miRNAs.

*B‐lymphocyte‐induced maturation protein 1* (*Blimp1*) regulates PGC specification by repressing *Hox* genes (Ohinata *et al.,* 2005; Vincent *et al.,* 2005; Robertson *et al.,* 2007) and is predominantly targeted by let‐7 (West *et al.,* 2009). It is thought that suppression of *Blimp1* by let‐7 upregulates *Hox* genes, leading totipotent PGCs towards male germline specification. Migration of PGCs to the female gonad is mediated by the miR‐17‐92 and miR‐290‐295 cluster, and the loss of this cluster disrupts PGC colonization in the female gonad, resulting in premature ovarian failure (POF) and sterility (Hayashi *et al.,* 2008; Medeiros *et al.,* 2011). The impact of PGC loss is much more severe in females than in males as female germline stem cells cannot propagate and self‐renew during adulthood, while spermatogonial stem cells (SSCs) successfully propagate and self‐renew throughout the lifetime, thus the loss of these miRNAs results in infertility in females but only subfertility in males.

miR‐22 suppresses *oestrogen receptor 1* (*ESR1*)‐mediated oestrogen signalling in ovine foetal testes, which is necessary for male gonad establishment (Torley *et al.,* 2011). Expression of miR‐202‐5p, miR‐202‐3p, miR‐140‐3p, and miR‐140‐5p is upregulated in committing testes and may mediate testis development as well as Leydig cell formation (Eggers, Ohnesorg, & Sinclair, 2014). By contrast, *SRY‐box 9* (*Sox9*) suppression is required for female gonad commencement (Eggers *et al.,* 2014); in its absence, sex reversal can occur (Eggers *et al.,* 2014). Thus, *Sox9* regulatory miR‐124 plays a key role in female gonad establishment (Eggers *et al.,* 2014). let‐7, miR‐23b, and miR‐21 regulate male PGC differentiation by mediating *Lin‐28 homolog A* (*Lin28)*/*let‐7*/*Blimp1* signalling (Brieno‐Enriquez *et al.,* 2015). In mice, the abundance of miR‐485 and miR‐878‐5p changes in a time‐dependent manner in the ovary and testes and may be crucial for sex specification (Rakoczy *et al.,* 2013). Similarly unique and differential expression of miRNAs is reported in the testes (Watanabe *et al.,* 2006; Luo *et al.,* 2010; Yang *et al.,* 2013a; Luo *et al.,* 2015b) and ovaries (Choi *et al.,* 2007; Liang *et al.,* 2007; Ahn *et al.,* 2010; Nothnick, 2012; Xiao *et al.,* 2014; Li *et al.,* 2015) as well as other reproductive organs and tissues including testicular somatic cells (Rakoczy *et al.,* 2013), epididymis (Belleannee *et al.,* 2012; Chu *et al.,* 2015; Nixon *et al.,* 2015a, 2015b), fallopian tube (Nothnick, 2012), uterus (Nothnick, 2012) and cervix (Nothnick, 2012) of different mammalian species. Several miRNAs (such as let‐7, miR‐503, miR‐672, miR‐465, miR‐21‐5p and miR‐143‐3p) could play a housekeeping role (Ahn *et al.,* 2010; Li *et al.,* 2015) and the miR‐34 family can be considered as a fertility biomarker (Tscherner *et al.,* 2014) in both sexes. Clearly, imbalances in miRNA regulation at any stage can disrupt the whole process of gonad specification and sex commitment and may cause sexual disorders as well as sex reversal of embryos.

## IV. ROLES OF miRNA IN MALE REPRODUCTION

A range of miRNAs are male germ cell stage‐specific and are predominantly detected in male reproductive tissues (Fig. 2), with impaired miRNA expression causing reduced testes size, degenerated seminiferous tubules, impaired spermatogenesis, increased germ cell apoptosis, and the production of abnormal spermatozoa (fragmented acrosomes, impaired chromatin compaction, deformed sperm head, folded tail, immotile sperm), and a decrease in the number of mature spermatozoa, leading to infertility (Table S2).

### (1) The formation of male gonads

Most miRNAs that are highly expressed in male PGCs are also detected in spermatogonia cells. Validated targets of these miRNAs are involved in *mitogen‐activated protein kinase* (*MAPK*), *janus kinase* (*JAK*)‐*signal transducer and activator of transcription proteins* (*STAT*), and *transforming growth factor beta* (*TGFβ*) signalling pathways (Wu *et al.,* 2014; Garcia‐Lopez *et al.,* 2015). A panel of miRNAs was detected in human and mouse testes (Watanabe *et al.,* 2006). Among these miRNAs, miR‐449a is abundant in both mouse and human testes (Luo *et al.,* 2015b). In boar testes, a subset of miRNAs are expressed differentially between mature and immature testes (Luo *et al.,* 2010) and regulates stage‐specific testicular gene expression. For example, miR‐34b and miR‐34c regulate the differential expression of *AQN‐1 protein* (*AQN‐1*), *hyaluronan synthase 3* (*HAS3*), *ring finger protein 4* (*RNF4*), *sperm mitochondria‐associated cysteine‐rich protein* (*SMCP*), and *sperm adhesion molecule 1* (*SPAM1*) between mature and immature porcine testes (Luo *et al.,* 2010), indicating that these miRNAs play important roles in species‐ and stage‐specific male germ cell development. Similarly, the testicular somatic cells of fertile and infertile males express miRNAs differentially, for example miR‐202‐5p, a member of the let‐7 family, is selectively expressed in Sertoli cells of fertile males with normal germ cells, but not in the Sertoli cells of infertile males (Dabaja *et al.,* 2015). Previous studies also showed that miR‐202‐5p, which is a downstream mediator of the testis‐determining factor SOX9 (Wainwright *et al.,* 2013), is expressed in Sertoli cells in the early XY gonad (Rakoczy *et al.,* 2013) and its expression is up‐regulated again in the testis at later developmental stages (Ro *et al.,* 2007). These data indicate that miR‐202‐5p is closely associated with male gonad development.

### (2) The commitment and maintenance of SSCs

Differential expression of miRNAs is observed at different stages of development and maturation of male germline cells (Fig. 2) (Hayashi *et al.,* 2008). A group of these miRNAs regulating *phosphatase and tensin homolog* (*Pten*) and *Stat* signalling is predominantly downregulated in spermatogonia compared to PGCs, and thus enhances the expression of *Pten* and *Stat* levels in spermatogonia compared to PGCs (Garcia‐Lopez *et al.,* 2015). Likewise, members of the miR‐290‐295 cluster are pluripotency related, and are downregulated in spermatogonia compared to PGCs and gonocytes (Zheng *et al.,* 2011; McIver *et al.,* 2012b), the proliferation‐ and differentiation‐regulatory miR‐143‐3p is upregulated in mice spermatogonia compared to in PGCs (Garcia‐Lopez *et al.,* 2015), and miR‐293 is expressed differentially between gonocytes and spermatogonia (McIver *et al.,* 2012b). This indicates that these miRNAs may specifically participate in the commitment of PGCs to SSCs. Among the miRNAs involved in SSC maintenance, miR‐21 regulates the stemness, proliferation, and survival of SSCs by regulating *ETS variant 5* (*ETV5*)/*glial cell‐derived neurotrophic factor* (*GDNF*) signalling (Niu *et al.,* 2011). GDNF is a well‐defined paracrine factor that promotes SSCs self‐renewal (Yang & Oatley, 2014), and is closely connected to the *ret proto‐oncogene* (*RET*) that is required for *GDNF family receptor alpha 1* (*GFRα1*) signalling in mouse SSCs (Jing *et al.,* 1996; Naughton *et al.,* 2006). With the assistance of the *phosphoinositide 3‐kinase* (*PI3K*)/*AKT serine/threonine kinase* (*AKT*)‐dependent pathway or sarcoma (Src) family kinase pathway, GDNF regulates the expression of *ETV5*, *inhibitor of DNA binding 4* (*ID4)*, *LIM homeobox 1* (*LHX1*), and *POU class 3 homeobox 1* (*POU3F1*) to drive SSC self‐renewal (Lee *et al.,* 2007; Oatley, Avarbock, & Brinster, 2007). Thus, miR‐21 appears to have a very important role in PGCs self‐renewal and maintenance.

Similarly, miR‐221/222 promotes the stemness characteristics of SSCs by downregulating *KIT proto‐oncogene receptor tyrosine kinase* (*c‐Kit*) expression in mice (Yang *et al.,* 2013b), and both miR‐20 and miR‐106a directly target *signal transducer and activator of transcription 3* (*Stat3*) and *cyclin D1* (*Ccnd*1), and play critical roles in maintenance and renewal of SSCs by increasing *proliferating cell nuclear antigen* (*Pcna*) and *promyelocytic leukaemia zinc finger* (*Plzf*), and reducing *c‐Kit* expression in mice (He *et al.,* 2013). miR‐290 and miR‐302 are also involved in the maintenance and pluripotency of SSCs in mice (Zovoilis *et al.,* 2008). It is well known that *miR‐135a and miR‐544 are essential factors for the* regulation of self‐renewal of SSCs in rat and goat, respectively (Moritoki *et al.,* 2014; Song *et al.,* 2015). In addition, miR‐184 and *miR‐204* are involved in mouse and goat SSC proliferation (Wu *et al.,* 2011), and protocadherins, which are also important cell‐adhesion molecules, are also targeted by miR‐320 during germ cell biogenesis (Marcon *et al.,* 2008). Differential expression of numerous miRNAs occurs between early and late stages of mammalian spermatogonia; these miRNAs are involved in either the maintenance or differentiation of SSCs (Niu *et al.,* 2011; Tong *et al.,* 2011, 2012; He *et al.,* 2013; Yang *et al.,* 2013b). Thus, growing evidence strongly suggests that many miRNAs are closely linked to the post‐transcriptional regulation of SSCs self‐renewal and maintenance.

### (3) The differentiation of male SSCs to spermatocytes

A global shift in testicular miRNA expression is observed during the very early stages of spermatogenesis and differentiation of spermatogonia (Golestaneh *et al.,* 2009). For example, miR‐146 downregulates *mediator complex subunit 1* (*Med1*), *c‐Kit*, and *spermatogenesis and oogenesis specific basic helix‐loop‐helix 2* (*Sohlh2*), and mediates the differentiation of SSCs (Huszar & Payne, 2013). *c‐Kit* is required for the differentiation of SSCs and is a target of miR‐221 and miR‐222 (Yang *et al.,* 2013b). Thus, suppression of miR‐221 and miR‐222 may promote the differentiation of SSCs. Also, miR‐34c promotes SSC differentiation in mice by suppressing *nanos C2HC‐type zinc finger 2* (*Nanos2*) and simultaneously upregulating *stimulated by retinoic acid 8* (*Stra8*) and *synaptonemal complex protein 3* (*Scp3*) (Yu *et al.,* 2014). In addition, members of the miR‐34 family regulate *notch 1* (*NOTCH1)*, *notch 2* (*NOTCH2)*, *TGFB‐induced factor homeobox 2* (*TGIF2*), *cyclin dependent kinase 4* (*CDK4*), and *MYC proto‐oncogene*, *BHLH transcription factor* (*c‐MYC*) (Yan *et al.,* 2009; Bouhallier *et al.,* 2010), and miR‐449 regulates *NOTCH1* and *B‐cell lymphoma 2* (*BCL2*) (Yan *et al.,* 2009); the genes together regulate the survival and differentiation of SSCs through Notch and *Tgfβ* signalling in mammals (Damestoy *et al.,* 2005; Kostereva & Hofmann, 2008; Yan *et al.,* 2009). *High mobility group AT‐hook 2* (*Hmga2*) and *outer dense fiber of sperm tails 1* (*ODF1*) are involved in the structural organization and differentiation of germ cells in mice and human, respectively (Chieffi *et al.,* 2002; Zhao *et al.,* 2007), and are targeted by the let‐7 family and miR‐22, respectively (Curry, Safranski, & Pratt, 2011). The let‐7 family also targets *insulin like growth factor 1* (*Igf1*) and may control spermatogonial differentiation in mice through *extracellular signal‐regulated kinase 1/2* (*Erk1/2*) and PI3K signalling (Shen *et al.,* 2014). miR‐383 regulates mitotic division in spermatogonia and primary spermatocytes by targeting *interferon regulatory factor 1* (*IRF1*), and subsequently downregulating *CCND1*, cyclin dependent kinase 2 (*CDK2*), and *cyclin dependent kinase inhibitor 1A* (*p21*) (Lian *et al.,* 2010; Luo, Hou, & Yang, 2016). Additionally, miR‐294 and miR‐295 are specific to SSCs, while miR‐201 and miR‐547 are specific to premeiotic male germ cells (Smorag *et al.,* 2012). This indicates that these miRNAs regulate differentiation of SSCs by controlling mainly *NOTCH*, *Erk1/2* and *PI3K* signalling.

Transition of B‐spermatogonia to spermatocytes accompanies major alterations in miRNA profiles (Liu *et al.,* 2015), and the deregulated miRNAs regulate the differential expression of *E2F transcription factor 1* (*E2F1*), *ETS variant 1* (*ETV1*), *TNF alpha‐induced protein 8 like 2* (*TNFAIP8l2*), *target of Myb1 membrane trafficking protein* (*TOM1*), *transforming growth factor beta receptor 1* (*TGFBR1*), and *bone morphogenetic protein receptor type 2* (*BMPR2*) between spermatogonia and spermatocytes (Liu *et al.,* 2015), potentially regulating apoptosis, proliferation, differentiation, junctional assembly, and cell cycle during spermatogenesis (Luo *et al.,* 2015a). For example, *methyl‐CpG binding domain protein 6* (*MBD6*) and *H2A histone family member X* (*H2AX*) are important for meiotic division during spermatogenesis and are putative targets of miR‐24 (McIver *et al.,* 2012a). *c‐Kit*, *RNA binding motif protein 44* (*Rbm44*), and *cyclin dependent kinase 6* (*Cdk6*) play roles in SSC maintenance and meiotic differentiation in mice and are regulated by miR‐221, miR‐203, and miR‐34b‐5p, respectively (Smorag *et al.,* 2012). In the early stages of male germ cell specification in mice, *cyclin T2* (*Ccnt2*) is regulated by miR‐15a, which is expressed stage specifically and time dependently during spermatogenesis (Teng *et al.,* 2011). miR‐15b targets *GDP dissociation inhibitor 1* (*GDI1*) and *isocitrate dehydrogenase 3 (NAD (+)) alpha* (*IDH3A*), and regulates TCA‐cycle mediated energy metabolism (Curry *et al.,* 2011). *Heat shock transcription factor 2* (*Hsf2*), an important molecule required for mammalian spermatogenesis, is regulated by miR‐18 during spermatogenesis in mice (Bjork *et al.,* 2010). *miR‐18*, which belongs to the *miR‐17‐92* cluster, is abundantly expressed in spermatocytes. Finally, *miR‐34b‐5p* regulates meiotic progression by targeting *Cdk6 (Smorag et al.,* 2012 *)*. Taken together, miRNAs are important regulators of cell cycle progression and differentiation during the transition of B‐spermatogonia to spermatocytes.

### (4) Meiotic sex chromosome inactivation (MSCI) and chromosomal stability during spermatogenesis

MSCI refers to the transcriptional silencing of genes located on the X‐chromosome at the pachytene spermatocyte stage. While most X‐chromosomal genes are silenced, X‐chromosome linked miRNAs including miR‐718‐3p, miR‐883a‐3p, and miR‐883a‐5p can escape MSCI‐mediated silencing in mice (Song *et al.,* 2009). This exceptional event allows these miRNAs to remain active throughout spermatogenesis and play a crucial role in the differentiation of SSCs (Song *et al.,* 2009). miRNAs are fine‐tuners of many important genes, and thus their absence can cause overexpression of X‐linked genes that are normally silenced by MSCI (Greenlee *et al.,* 2012), ultimately leading to the impairment of meiotic progress, acrosomal defects, abnormal chromatin condensation and nuclear shaping, degenerated seminiferous tubules, mixing of different stages of haploid spermatogonia, and the presence of exfoliated and immature germ cells in the epididymis (Korhonen *et al.,* 2011), followed by infertility in males (Greenlee *et al.,* 2012). Although miRNAs such as miR‐871‐3p, miR‐743‐3p, and miR‐883b‐3p may regulate MSCI‐mediated gene expression (Song *et al.,* 2009), but there is not yet direct evidence for this. miRNAs are also important for maintaining chromosomal stability; the loss of several miRNAs such as miR‐16, miR‐18, miR‐183, and miR‐762 caused abundant activation of *ATM serine/threonine kinase* (*ATM*) and deregulation of genes including *mediator of DNA damage checkpoint 1* (*Mdc1*), *Cdk2*, *trimethylation of lysine 9 on histone H3* (*H3K9me3*), and *ring finger protein 8* (*Rnf8*), which are involved in chromosomal stability and DNA repair during spermatogenesis in mice (Modzelewski *et al.,* 2015). miR‐762 downregulates DNA damage‐related gene *H2A histone family member X* (*γ‐H2AX*) and upregulates the DNA damage repair‐related genes *RNF8*, *ring finger protein 168* (*RNF168*), *DNA repair associated* (*BRCA1*), *tumor protein P53 binding protein 1* (*TP53BP1*), and *poly (ADP‐ribose) polymerase 1* (*PARP*), as well as promoting the growth of immature Sertoli cells in pigs (Ma *et al.,* 2016a), indicating that both miRNA‐mediated gene regulation and fine‐tuning of gene expression are critical factors for MSCI and chromosomal stability during spermatogenesis.

### (5) miRNAs as regulators of spermiogenesis and chromatin body compaction

A wide range of miRNAs are expressed differentially during the transition of pachytene spermatocytes to round spermatids and might be important in the spermiogenesis process. For example, *round spermatid basic protein 1* (*Rsbn1*) is an important molecule for the transcriptional regulation of haploid germ cell formation and is a target gene of miR‐355, miR‐181b, and miR‐181c (Yan *et al.,* 2007; McIver *et al.,* 2012a). In addition, miR‐122 downregulates *transition protein 2* (*Tnp2*) (Yu, Raabe, & Hecht, 2005), which replaces protamine, an essential component for the compaction and patterning of chromatin bodies into small sperm heads (Krawetz *et al.,* 2011); testis‐specific miR‐469 also plays an important role in chromatin remodelling and elongation of spermatids by repressing *Tnp2* and *protamine 2* (*Prm2*) (de Mateo & Sassone‐Corsi, 2014). Similarly, loss of miRNA‐dependent regulation of *Arpc5* impairs translational activation of *protamine 1* (*Prm1*)/*Prm2* by blocking 80S ribosome formation and facilitating mRNA transportation to chromatoid/P bodies in mice (Chang *et al.,* 2012). miRNA‐dependent regulation of *tudor domain containing 6* (*Tdrd6*) during spermiogenesis of mice is essential for chromatin body formation (Vasileva *et al.,* 2009). However, unlike the early stages of spermatogenesis, most of these miRNAs disappear during spermiogenesis, leaving a few sperm‐borne miRNAs such as *miR‐34 to regulate* fertilization and early zygote development (Liu *et al.,* 2012). Therefore, some of the sperm‐borne miRNAs are important epigenetic contributors to the embryo. However, the roles of miRNAs in chromatin compaction require further investigation to determine whether the impairments caused by deregulated miRNAs are permanent or recoverable.

### (6) miRNAs regulate maturation and viability of spermatozoa during storage and transportation through the epididymis

Epididymal spermatozoa are enriched with distinctive miRNAs (Fig. 2), which are uncommon in other spermatozoal stages (Nixon *et al.,* 2015b; Reilly *et al.,* 2016). Additionally, epididymal epithelial cell‐derived miRNAs may influence the post‐testicular progression of sperm to their destination and mediate cell‐to‐cell communication (Belleannee *et al.,* 2013a; Belleannee, 2015; Qing *et al.,* 2017); vasectomy results in alteration of miRNAs in both epididymal and seminal microvesicles (Belleannee *et al.,* 2013b). Differential expression of miRNAs is also found between epididymal and freshly ejaculated sperm, as well as among the segments (caput, corpus, cauda) of the epididymis in different species including human (Belleannee *et al.,* 2012), horse (Twenter *et al.,* 2017), mouse (Nixon *et al.,* 2015a,*b*), pig (Chang *et al.,* 2016) and bull (Belleannee *et al.,* 2013a). These miRNAs might be involved in spermatozoa maturation (Twenter *et al.,* 2017), intracellular trafficking signalling (Nixon *et al.,* 2015a), death signalling (Chu *et al.,* 2015), and epididymal cell proliferation (Ma *et al.,* 2012). The differential expression of miRNAs among the epididymal segments is common to various mammalian species, thus it could be assumed that the contribution of epididymal miRNAs is an essential part of mammalian sperm maturation and concentration, and that these epididymal miRNAs potentially contribute to the motility of spermatozoa as well as to the content of the seminal fluid.

### (7) miRNAs are potent regulators of male germline cell apoptosis in mammals

Apoptosis in spermatocytes is a common event as meiotic cells have to undergo homologous recombination through double‐strand breaks (Hamer *et al.,* 2003). Several miRNAs such as the miR‐449 cluster and miR‐34b/c, regulate male germ cells by targeting the E2F‐retinoblastoma protein (pRb) pathway in the early meiotic phase (Bao *et al.,* 2012). In male sheep with disrupted spermatogenesis, the expression of miR‐144 (homolog miR‐98) was upregulated; miR‐144 regulates the apoptosis‐related genes *fas ligand* (*FASL*), *caspase 3* (*CAS3*), *tumor protein 53* (*TP53*), and *BCL2‐like 1* (*BCL2L1*) (Guan *et al.,* 2015). Furthermore, miR‐26a induces G_1_ arrest by regulating *cyclin E1* (*CCNE1*), *cyclin E2* (*CCNE2*), *cyclin D2* (*CCND2*), and *CDK6* (Kota *et al.,* 2009) and then induces apoptosis in parallel with the direct effects of miRNA‐144. Similarly, changes in the expression of miRNA‐99a seem to affect the organization of Sertoli‐cell tight junctions by targeting *zona occludens 1* (*ZO‐1*) (Turcatel *et al.,* 2012), whereas miR‐34c enhances germinal phenotypes in late spermatogenesis (Bouhallier *et al.,* 2010).

In oestradiol benzoate‐treated rats, the miR‐29 family resulted in extensive apoptosis of male germ cells by downregulating *Dnmt* and *myeloid cell leukemia 1* (*Mcl‐1*) (Meunier *et al.,* 2012). Ochratoxin‐A treatment induces extensive apoptosis of male germ cells, while inhibition of miR‐122 minimizes this Ochratoxin‐A‐mediated toxicity (Chen *et al.,* 2015a), indicating that miR‐122 is a critical regulator for the germ cell apoptosis‐induction pathway. In pigs, expression of miR‐16 and miR‐34 regulates the cell cycle progression during spermatogenesis and senescence of sperm (Luo *et al.,* 2015b). miR‐16 promotes the apoptosis of immortalized GC‐1 spermatogonia cells (GC‐1 SPG) by downregulating *Ccnd1* (Li *et al.,* 2016b). Deletion of the miR‐17‐92 cluster, which is potentially regulated by transcriptional regulation of *c‐MYC* and *E2F1* (Novotny *et al.,* 2007), results in testicular atrophy and empty seminiferous tubules by reducing SSCs and increasing germ cell apoptosis (Xie *et al.,* 2016). Taken together, these observations suggest that deregulation in miRNA expression is an important mechanism behind mammalian male germline cell apoptosis.

## V. ROLES OF miRNAs IN FEMALE REPRODUCTION

Sex‐specific and unique miRNA profiles are observed at every step of female reproduction (Fig. 2): female‐specific miR‐124 promotes the commitment of female gonads and prevents sex reversal by silencing *Sox9* in the mouse ovary. Similarly, many miRNAs play substantial roles throughout folliculogenesis and oogenesis by regulating expression of important genes (see Table S3), and many are specific to particular developmental stages of the follicles, as well as segments of the reproductive tract.

### (1) Growth and maturation of ovarian follicles

Folliculogenesis refers to the progression of small primordial follicles into large preovulatory follicles that occurs in part during the oestrus cycle. During folliculogenesis, the majority of follicles commit to atresia, and a few develop into Graafian follicles. miR‐145 regulates initiation of the growth, development, and maintenance of mouse primordial follicles (Fig. 2) by regulating *Tgfβ* signalling including *transforming growth factor beta receptor 2* (*Tgfbr2*), *activin A receptor type 1B* (*Acvr1b*), *SMAD family member 3* (*Smad3*), and *SMAD family member 5* (*Smad5*) (Yang *et al.,* 2013c), while loss of miR‐145 over‐activates primordial follicles and deregulates zona pellucida in growing follicles (Yang *et al.,* 2013c). Similarly, miR‐224 enhances folliculogenesis in mice by targeting *SMAD family member 4* (*Smad4*) and regulating *transforming growth factor beta 1* (*Tgfβ1*)‐induced proliferation of granulosa cells (GCs) (Yao *et al.,* 2010a), and miR‐133b regulates the development of ovaries and maturation of oocytes by targeting *transgelin 2* (*TAGLN2*), an actin protein‐encoding gene (Xiao *et al.,* 2014). In porcine oocytes, miR‐205 participates in *brain‐derived neurotrophic factor* (*BDNF*)‐induced maturation by targeting *pentraxin 3* (*PTX3*) (Li *et al.,* 2016a) and miR‐29 has a role during the development of bovine follicles (Hossain *et al.,* 2009).

Numerous miRNAs play a role in growing follicles (Fig. 2) (Lei *et al.,* 2010; Yao *et al.,* 2010a; Xu *et al.,* 2011b; Sen *et al.,* 2014), selection of dominant follicles (Salilew‐Wondim *et al.,* 2014), formation of germinal vesicle (GV) oocytes (Murchison *et al.,* 2007; Tripurani *et al.,* 2010), maturation of oocytes (Tesfaye *et al.,* 2009; Abd El Naby *et al.,* 2013; Xiao *et al.,* 2014; Song *et al.,* 2016; Li *et al.,* 2016a), ovulatory follicles (Carletti, Fiedler, & Christenson, 2010; Sontakke *et al.,* 2014; Wright *et al.,* 2016), the ovulation process (Fiedler *et al.,* 2008; Hasuwa *et al.,* 2013; Ahmed *et al.,* 2017), and development of metaphase II (M‐II) oocytes (Xu *et al.,* 2011b; Wright *et al.,* 2016). Among these miRNAs, miR‐202 is gonad‐specific and might be important in the prevention of premature ovarian failure (POF) (Sontakke *et al.,* 2014), and miR‐27b and its target, *peroxisome proliferator activated receptor gamma* (*PPARγ*), is crucial for the maturation of porcine oocytes (Song *et al.,* 2016). In humans, miR‐15a potentially regulates the growth and maturation of oocytes by regulating *BCL2* and *cell division cycle 25A* (*CDC25A*) (Xu *et al.,* 2011b), while miR‐335‐5p regulates spindle formation and cytoskeleton dynamics *via* MAPK signalling in mouse oocytes (Cui *et al.,* 2013). These data indicate that each stage of folliculogenesis has a unique profile of miRNA expression and that miRNAs are involved in the selection, growth, development, and maturation of ovarian follicles.

### (2) miRNA expressions varies in the granulosa cells (GCs) and thecal cells (TCs) of the dominant follicles (DFs) and subordinate follicles (SFs)

DFs and SFs show differential expression of some miRNAs, which may regulate *gonadotropin‐releasing hormone* (*GnRH*) signalling (Salilew‐Wondim *et al.,* 2014). These miRNAs are expressed differentially among different subgroups of GCs (Tesfaye *et al.,* 2009), TCs (Zielak‐Steciwko *et al.,* 2014), and between mural and cumulus cells (Velthut‐Meikas *et al.,* 2013). These miRNAs are also potential regulators of numerous signalling pathways including meiosis, *wingless‐type MMTV integration site family* (*WNT*), *TGFβ*, *ErbB receptor tyrosine kinases* (*ERBB*), *insulin*, *P13K–AKT*, and *MAPK* (Zielak‐Steciwko *et al.,* 2014). Another panel of miRNAs including miR‐125b, miR‐145, and miR‐199a‐3p target *leukemia inhibitory factor* (*LIF*), *cyclin dependent kinase inhibitor 1A* (*CDKN1A*), and *prostaglandin‐endoperoxide synthase 2* (*PTGS2*), respectively, in GCs, but not in TCs of bovine follicles (Donadeu *et al.,* 2012). Furthermore, a panel of miRNAs is highly expressed in atretic follicles compared to healthy follicles, with all miRNAs except miR‐21‐3p and miR‐378 showing higher expression in TCs compared to GCs (Donadeu, Mohammed, & Ioannidis, 2017). This suggests that these miRNAs might regulate the phenotypic characters of different cell types by controlling the expression of important signalling molecules (Donadeu *et al.,* 2017). Additionally, miR‐708, miR‐221, miR‐21‐3p, miR‐335, and miR‐214 are expressed abundantly in the follicular fluid of SFs compared to that of DFs (Gebremedhn *et al.,* 2015) indicating a potential role in the selection of follicles. Hence, differential expression of miRNAs between TCs and GCs may play an important role in the selection and recruitment of DFs, as well as during the follicular wave (in different mammalian species the recruitment, growth and development of several waves of follicles is possible before follicle rupture and ovulation occur; during this process two or more follicles might develop, but only one follicle successfully releases the egg) and atresia.

### (3) Atresia and degeneration of ovarian follicles

miRNAs are potent regulators of follicular atresia and degeneration (Fig. 2) (Lin *et al.,* 2012; Cao *et al.,* 2015; Chen *et al.,* 2015b). In brief, the apoptosis of human GCs is regulated by miR‐146a, which directly targets *interleukin 1 receptor associated kinase 1* (*IRAK1*) and *TNF receptor associated factor 6* (*TRAF6*), and indirectly regulates *nuclear factor kappa B* (*NFκB*), *phospho‐inhibitory subunit of NF kappa B alpha* (*pIκBα*), *caspase 8* (*CAS8*), *caspase 9* (*CAS9*), *CAS3*, and *PARP* (Chen *et al.,* 2015b). Follicular atresia in pigs is regulated by members of the let‐7 family, which regulate *MAPK*, *TGFβ*, and *p53* signalling pathways; all other members are downregulated in atretic and progressively atretic follicles except let‐7 g (Cao *et al.,* 2015). In addition, miR‐26b targets *ATM* and enhances GC apoptosis during follicular atresia in pigs (Lin *et al.,* 2012). miR‐181a, which is highly expressed in primary follicles over preantral and antral follicles and leads to POF (Zhang *et al.,* 2013b) inhibits the production of GCs and follicular development in mice by suppressing *Acvr2a* and decreasing phosphorylation of *Activin*‐signalling. Similarly, miR‐143 inhibits the proliferation of pregranulosa cells in mice by regulating *Cdks*, *Ccnb1*, *Ccnd2*, and *Ccne2*, and thus inhibits the formation of primordial follicles (Zhang *et al.,* 2013a). In cattle, miR‐21‐5p, miR‐21‐3p, miR‐222, miR‐155, and miR‐199a‐5p are upregulated in atretic follicles compared to healthy follicles (Donadeu *et al.,* 2017). Another two miRNAs, miR‐378 and miR‐21‐5p, were increased in subordinate and anovulatory follicles in horses (Donadeu & Schauer, 2013; Schauer *et al.,* 2013). Of note, miR‐21 prevents follicular atresia and regulates follicle survival, follicular to luteal transition, and ovulation (Carletti *et al.,* 2010). Androgen‐induced miR‐125b suppresses the pro‐apoptotic genes *BCL2 antagonist/killer 1* (*Bak*), *BCL2 associated X protein* (*Bax*), *Bcl2 modifying factor* (*Bmf*), and *TP53* in mouse GCs and promotes follicular survival by preventing atresia (Sen *et al.,* 2014). miR‐376a reduces oocyte apoptosis, increases primordial follicle number, and regulates follicular assembly by modulating *Pcna* in mice (Zhang *et al.,* 2014). Therefore, miRNAs appear to have a very important regulatory role during apoptosis of GCs and follicular atresia.

### (4) Regulation of luteinization and formation of the CL

The importance of miRNA signalling for regulation of the CL has been demonstrated using hypomorphic *Dicer1* mutant mice, which fail to implant embryos and maintain pregnancy due to impaired growth of new capillary vessels in the CL (Otsuka *et al.,* 2008). The study demonstrated that the impaired CL angiogenesis in *Dicer1* (d/d) mice was associated with a lack of miR‐17‐5p and let‐7b, which target *tissue inhibitor of metalloproteinases 1* (*Timp1)*. These data suggest that the pro‐angiogenic miR‐17‐5p and let‐7b are critical factors for the normal development of the CL. miRNAs are known to be involved in the process of CL formation (Fig. 2) such as during follicular to luteal transition (McBride *et al.,* 2012), vascularization of luteal tissue (Otsuka *et al.,* 2008), and in non‐regressed CL (Ma *et al.,* 2011), and regressed CL (Ma *et al.,* 2011). In particular, reduction of miR‐503 expression in pre‐ovulatory follicles, signals the onset of luteinization, with its expression subsequently restored during CL development (Lei *et al.,* 2010; McBride *et al.,* 2012). miR‐503 directly regulates *Gdf9*, *follicle stimulating hormone Receptor* (*Fshr*), *oestrogen receptor 2* (*Esr2*), *activin A receptor type 2A* (*ActRIIa*), *Ccnd2*, and *activin A receptor type 2B* (*ActRIIb*) and indirectly regulates *inhibin alpha subunit* (*Inha*), *Inhba*, *Inhbb*, *Cyp19a1*, *luteinizing hormone/choriogonadotropin receptor* (*Lhcgr*), *Esr2*, *androgen receptor* (*Ar*), *Cdkn1b*, and *Cas3* (Lei *et al.,* 2010). Thus, miR‐503 could have a direct involvement in CL formation and maintenance. Another study showed that miR‐378 plays an anti‐apoptotic role in luteal cells of non‐regressed CL by targeting *interferon gamma receptor 1* (*IFNGR1*) (Ma *et al.,* 2011). These observations provide evidence to explain the physiological requirement for miRNAs during luteal development.

### (5) The prediction of oocyte quality

miRNAs are expressed abundantly in follicular fluid, cumulus cells, and oocytes. Therefore, it may be possible to use miRNAs to evaluate the quality of oocytes. Uhde *et al*. (2017) attempted to assess the quality of bovine oocytes based on the miRNA expression of the surrounding cumulus cells although they concluded that the quality of pre‐fertilization‐stage oocytes could not be determined in this way. As highlighted in Fig. 2, the functions of miRNAs are globally suppressed between matured oocytes and first zygotic cell division stages (Suh *et al.,* 2010). As a result, the functional activity of miRNAs are generally abrogated (although the expression of miRNAs remains abundant) during those stages (Tang *et al.,* 2007; Suh *et al.,* 2010) representing an obstacle to the use of miRNAs as biomarkers to assess the quality of pre‐fertilization‐stage oocytes. However, the roles of maternal miRNAs (oocyte‐derived miRNAs) are indispensable during the post‐fertilization stages (Tang *et al.,* 2007; Karakaya *et al.,* 2015), especially during and after zygotic genome activation. It may be that the roles of oocyte‐derived miRNAs will be understood only by monitoring the post‐fertilization development potential of oocytes.

### (6) miRNAs and extracellular vesicles within follicular fluid

Extracellular vesicles including exosomes are tiny particles of biological origin, contain RNAs, proteins, lipids and other small molecules, and are transported by the circulatory system to neighbouring or distant organs (da Silveira *et al.,* 2012; Reza *et al.,* 2016). These particles function as a cargo transport system and participate in cell–cell communication, embryo–maternal crosstalk (Saadeldin, Oh, & Lee, 2015) and communication among ovarian follicles (da Silveira *et al.,* 2012). Vesicular miRNAs derived from follicular fluid are being investigated for their suitability as biomarkers for oocyte quality in matured and immature oocytes (Sohel *et al.,* 2013; Santonocito *et al.,* 2014; da Silveira *et al.,* 2015), as well as in healthy and unhealthy follicles (Sang *et al.,* 2013; Roth *et al.,* 2014). For example, matured follicle‐derived microvesicles are enriched with a panel of miRNAs including miR‐99a, miR‐100, miR‐132 and miR‐218 (Santonocito *et al.,* 2014), among which miR‐132, miR‐212 and miR‐214 negatively regulate the factors related to the inhibition of follicular maturation while miR‐29a is involved in epigenetic changes (Santonocito *et al.,* 2014). In addition, follicular‐fluid‐derived circulatory miRNAs are potentially involved in steroidogenesis processes (Sang *et al.,* 2013), for example, miR‐132, miR‐320, miR‐520c‐3p, miR‐24 and miR‐222 regulate the concentration of oestradiol, while miR‐24, miR‐193b and miR‐483‐5p regulate the concentration of progesterone (Sang *et al.,* 2013). These data indicate that follicular‐fluid‐derived extracellular miRNAs could be an important tool to assess oocyte quality and to differentiate between healthy and unhealthy follicles.

## VI. miRNAs are potent regulators of the steroidogenic process during gametogenesis

**Figure 3:**
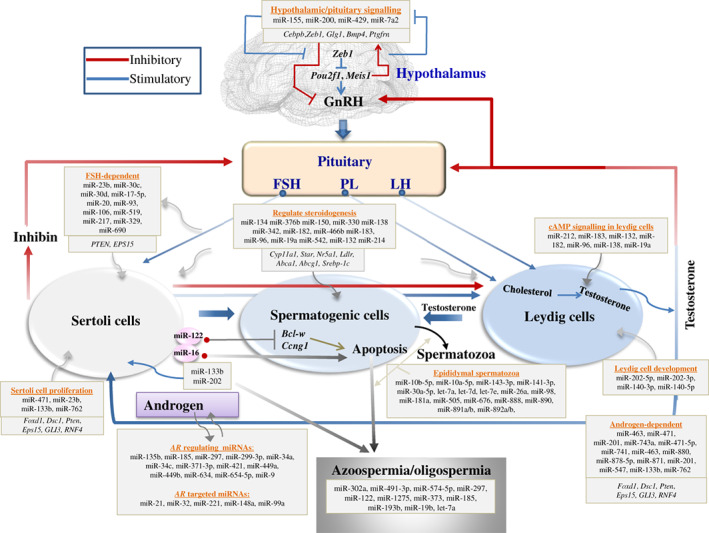
MicroRNA (miRNA) regulation in the maintenance of male hormonal balance. A panel of miRNAs regulates *Pou2f1*‐ and *Meis1*‐mediated gonadotropin‐releasing hormone signalling by targeting *Zeb1*, and thus these miRNAs may play important roles in the maintenance of hormonal balance. The expression of miRNAs changes with levels of different reproductive hormones; additionally, hormonal balance is reported to be miRNA‐dependent, as miRNAs regulate the proliferation, differentiation, function, and apoptosis of male steroidogenic cells. Thus, miRNAs, genes, and hormones develop a feedback loop that optimizes spermatogenesis; any imbalance in the feedback loop can result in azoospermia, oligospermia, infertility, and reproductive failure in males. FSH, follicle‐stimulating hormone; LH, luteneizing hormone; PL, prolactin; GnRH, gonadotropin‐releasing hormone; PTEN, phosphatase and tensin homolog; EPS15, epidermal growth factor receptor pathway substrate 15; AR, androgen receptor; Cebpb, CCAAT/enhancer binding protein beta; Zeb1, zinc finger E‐box binding homeobox 1; Glg1, golgi glycoprotein 1; BMP4, bone morphogenetic protein 4; Ptgfrn, prostaglandin F2 receptor inhibitor; Pou2f1, POU class 2 homeobox 1; Meis1, meis homeobox 1; Cyp11a1, cytochrome P450 family 11 subfamily A member 1; Star, steroidogenic acute regulatory protein; Nr5a1, nuclear receptor subfamily 5 group A member 1; Ldlr, low density lipoprotein receptor; Abca1, ATP binding cassette subfamily A Member 1; Abcg1, ATP binding cassette subfamily G member 1; Srebp‐1c, sterol regulatory element binding transcription factor 1; Bcl‐w, BCL2 like 2; Ccng1, cyclin G1; Foxd1, forkhead box D1; Dsc1, desmocollin 1; GLI3, GLI family zinc finger 3; RNF4, ring finger protein 4.

**Figure 4:**
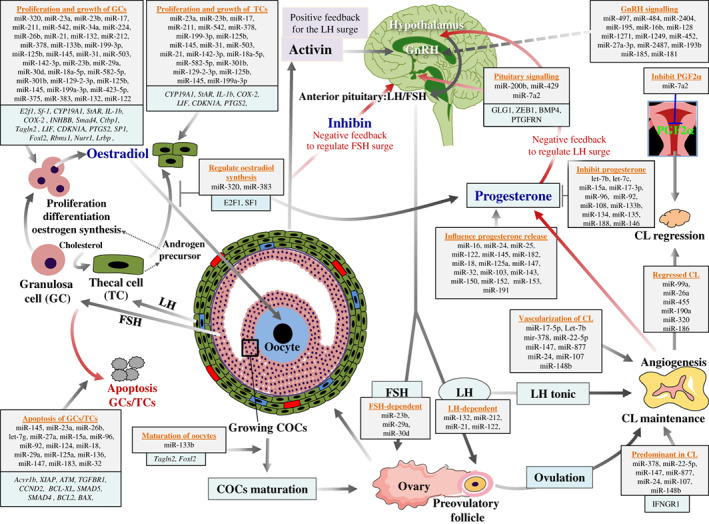
MicroRNA (miRNA) regulation in the maintenance of female hormonal balance. The proliferation, growth, differentiation, functioning, and apoptosis of female steroidogenic cells [granulosa cell (GC) and thecal cell (TC)] are highly regulated by miRNAs. miRNAs are also active regulators of female steroidogenic genes including c*ytochrome P450 family 19 subfamily A member 1* (*Cyp19a1*); *steroidogenic acute regulatory protein* (*Star*), and *prostaglandin‐endoperoxide synthase 2* (*Ptgs2*); thus, miRNAs directly regulate the steroidogenic process in females through gonadotropin‐releasing hormone (GnRH), follicle‐stimulating hormone (FSH), and luteinizing hormone (LH) signalling. The vasculogenesis and angiogenesis of progesterone‐producing corpus lutem (CL) is also related to the expression of miRNAs, making miRNAs important regulatory molecules during the female steroidogenic processes. miRNAs are involved in both follicular steroidogenesis and luteal steroidogenesis. PGF2α, prostaglandin; COC, cumulus‐oocyte complex; E2f1, E2F transcription factor 1; Sf‐1, steroidogenic factor 1 nuclear receptor; IL‐1b, interleukin 1 beta; COX‐2, cyclooxygenase 2; INHBB, inhibin beta B subunit; Ctbp1, C‐terminal binding protein 1; Tagln2, transgelin 2; LIF, leukemia inhibitory factor; CDKN1A, cyclin dependent kinase inhibitor 1A; SP1, specificity protein 1; Foxl2, forkhead box L2; Rbms1, RNA binding motif single stranded interacting protein 1; Nurr1, nuclear receptor subfamily 4 group A member 2; Lrbp, LH receptor mRNA‐binding protein; Acvr1b, activin A receptor type 1B; XIAP, X‐linked inhibitor of apoptosis; ATM, ATM serine/threonine kinase; TGFBR1, transforming growth factor beta receptor 1; CCND2, cyclin D2; BCL‐XL, BCL2 like 1; SMAD5, SMAD family member 5; SMAD4, SMAD family member 4; BCL2, B‐cell lymphoma 2; BAX, BCL2 associated X protein; GLG1, Golgi glycoprotein 1; ZEB1, zinc finger E‐box binding homeobox 1; BMP4, bone morphogenetic protein 4; PTGFRN, prostaglandin F2 receptor inhibitor; IFNGR1, interferon gamma receptor 1.

miRNAs are potent regulators of testicular, ovarian, hypothalamic and pituitary hormones (Figs 3 and 4) (Messina & Prevot, 2017), and regulate the expression and functions of numerous steroidogenic genes (see Table S4). Thus miRNAs act as an essential mediator to maintain interactions among the hypothalamus, pituitary, and gonad.

### (1) Hormonal balance and steroidogenesis in males

miRNAs and hormones together with genes develop an effective feedback loop to coordinate successful spermatogenesis (Fig. 3). The genes in the hypothalamus that regulate GnRH release and pituitary functions including *zinc finger E‐box binding homeobox 1* (*Zeb1*), *POU class 2 homeobox* (*Pou2f*), *meis homeobox 1* (*Meis1*), *golgi glycoprotein 1* (*Glg1)*, *bone morphogenetic protein 4* (*Bmp4*) and *prostaglandin F2 receptor inhibitor* (*Ptgfrn*), are effectively controlled by a panel of miRNAs including miR‐155, miR‐200, miR‐429 and miR‐7a2 (Hasuwa *et al.,* 2013; Ahmed *et al.,* 2017; Messina & Prevot, 2017). Therefore, these miRNAs might have direct or indirect control over the hormones released by the pituitary. Following downstream signalling from the pituitary gland, follicle‐stimulating hormone (FSH), luteinizing hormone (LH) and androgens become functional or inactivated, and this could stimulate the expression of different miRNAs in the testes (Panneerdoss *et al.,* 2012; Ma *et al.,* 2016a), leading to miRNA‐mediated regulation of growth, proliferation, maintenance and apoptosis of testicular cells (Nicholls *et al.,* 2011; Panneerdoss *et al.,* 2012; Shih *et al.,* 2015; Yao *et al.,* 2016; Ma *et al.,* 2016a). Alternatively, any deregulation of miRNAs in the testes could alter the expression of important genes that could disrupt the hormone balance, resulting in spermatogenesis failure, reproductive diseases and infertility such as azoospermia or oligospermia in males (Eggers *et al.,* 2014; Hu *et al.,* 2013; Shih *et al.,* 2015; Ghorbian, 2012).

In human, miR‐133b regulates Sertoli cell proliferation by directly targeting *GLI family zinc finger 3* (*GLI3*) following the upregulation of *CCNB1* and *CCND1* (Yao *et al.,* 2016). In pigs, miR‐762 promotes the growth and proliferation of immature Sertoli cells by targeting *RNF4* (Ma *et al.,* 2016a). In the mouse, miR‐471 directly participates in steroidogenesis by regulating *forkhead box D1* (*Foxd1*) and *desmocollin 1* (*Dsc1*): these genes regulate the metabolism of Sertoli cells and cell–cell adhesion in the testis, respectively (Panneerdoss *et al.,* 2012). In the rat, miR‐132 and miR‐214 repress *sterol regulatory element binding transcription factor 1* (*Srebp‐1c*) and *low density lipoprotein receptor* (*Ldlr*), respectively, and both regulate steroidogenesis (Hu *et al.,* 2013). miRNA‐23b regulates *Pten* and *epidermal growth factor receptor pathway substrate 15* (*Eps15*) in an FSH‐dependent manner, and its function is implicated in endocytosis, focal adhesion, and actin cytoskeleton regulation during spermatogenesis (Nicholls *et al.,* 2011). By contrast, numerous miRNAs in steroidogenic cells are expressed in an FSH‐ and androgen‐dependent manner (Nicholls *et al.,* 2011; Panneerdoss *et al.,* 2012; Shih *et al.,* 2015). This indicates that the expression of miRNAs and hormones are interdependent; a change in one of them can alter the expression of the other. It has been reported that patients suffering from azoospermia and oligospermia showed deregulated miRNA expression profiles (Ghorbian, 2012). Therefore, miRNAs and hormones potentially develop a feedback loop in the testes, and hormone‐mediated gene expression in the testes is regulated in a miRNA‐dependent manner.

### (2) Hormonal balance and steroidogenesis in females

miRNAs mainly regulate steroidogenesis in females by regulating the growth, proliferation, and apoptosis of female steroidogenic cells, GCs and TCs. In addition, hypothalamic GnRH signalling following changes in levels of FSH and LH is also regulated by a panel of miRNAs (Hasuwa *et al.,* 2013; Ahmed *et al.,* 2017; Messina & Prevot, 2017). The synthesis and inhibition of steroidogenic hormones including oestrogen, progesterone, and prostaglandin (PGF2α) are also regulated in a miRNA‐dependent manner. In humans, miR‐23a/b targets *CYP19A1* (Alford *et al.,* 2007), and miR‐17, miR‐211, and miR‐542 target *steroidogenic acute regulatory protein* (*StAR*), *interleukin 1 beta* (*IL‐1b*), and *cyclooxygenase 2* (*COX‐2*), respectively. Thus, these miRNAs regulate the steroidogenesis process in follicular cells (Toloubeydokhti, Bukulmez, & Chegini, 2008). In pigs, miR‐34a and miR‐26b target *INHBB* and *SMAD4*, respectively, and negatively regulate the steroidogenic process by promoting the apoptosis of GCs (Liu *et al.,* 2014; Tu *et al.,* 2014). Similarly, miR‐423‐5p and miR‐378 post‐transcriptionally downregulate *CYP19A1* (Xu *et al.,* 2011a; Sui *et al.,* 2014) and miR‐375 binds to the 3′ untranslated region of *specificity protein 1* (*SP1*) and mediates *corticotropin releasing hormone* (*CRH*) signalling (Yu *et al.,* 2017): these miRNAs directly regulate oestradiol synthesis in porcine GCs. In mice, miR‐224 enhances Tgfβ‐induced proliferation of GCs by targeting *Smad4* and promotes oestradiol biogenesis by increasing *Cyp19a1* (Yao *et al.,* 2010a). miR‐21 plays anti‐apoptotic roles and facilitates the maintenance of GCs, both *in vitro* and *in vivo* (Carletti *et al.,* 2010), while miR‐132 and miR‐212 regulate the differentiation of GCs by targeting *C‐terminal binding protein 1* (*Ctbp1*) (Fiedler *et al.,* 2008). miR‐132 regulates cyclic adenosine monophosphate (cAMP) signalling and promotes oestradiol production in mouse GCs through translational repression of *nuclear receptor related 1* (*Nurr1*) following upregulation of *Cyp19a1* (Wu *et al.,* 2015), and miR‐122 increases *LHCGR mRNA‐binding protein* (*Lrbp*) expression by activating *Srebp* in rat ovaries (Menon *et al.,* 2013).

Steroid‐producing GCs are also regulated by *TAGLN2*‐ and *forkhead box L2* (*FOXL2*)‐targeting miR‐133b (Dai *et al.,* 2013; Xiao *et al.,* 2014), which enhances *StAR* and *CYP19A1*, as well as oestradiol production (Dai *et al.,* 2013). Similarly, miR‐383 facilitates oestradiol biosynthesis by inhibiting *RNA binding motif single‐stranded interacting protein 1* (*Rbms1*), a DNA‐binding protein that activates *c‐Myc* in mouse GCs (Yin *et al.,* 2012), while the expression of miR‐383 is regulated by equine chorionic gonadotropin (eCG) and human chorionic gonadotropin (hCG) (Donadeu *et al.,* 2012). Additionally, FSH alters the expression of 31 miRNAs in rat GCs. Among them, miR‐23b is increased, whereas miR‐29a and miR‐30d are decreased after 12 h of exposure; however, both miR‐29a and miR‐30d were increased after 48 h of exposure (Yao *et al.,* 2010b), indicating that the length of hormonal exposure influences miRNA expression. Similarly, exposure of GCs to LH/hCG enhances the expression of miR‐132, miR‐212, miR‐21, and miR‐122 (Fiedler *et al.,* 2008; Carletti *et al.,* 2010; Menon *et al.,* 2013). In addition, 57 miRNAs including members of the let‐7 family, miR‐16, and miR‐122 play a role in female reproduction by inhibiting testosterone biosynthesis (Sirotkin *et al.,* 2009). miR‐320 also regulates the proliferation and functions of GCs by targeting *E2f1* and *splicing factor 1* (*Sf1*), thereby promoting progesterone and testosterone levels and inhibiting E2 biosynthesis in mice (Yin *et al.,* 2014). Similarly, 36 miRNAs including the let‐7 family, miR‐15a, and miR‐17‐3p inhibit progesterone biosynthesis, and 16 miRNAs including miR‐16, miR‐24, and miR‐191 promote progesterone synthesis in GCs (Sirotkin *et al.,* 2009). By contrast, miR‐107 enhances testosterone biosynthesis in female gonads (Sirotkin *et al.,* 2009). In addition, prenatal exposure to testosterone greatly influences the expression of miR‐497 and miR‐15b in sheep ovaries (Luense *et al.,* 2011).

By contrast, many miRNAs play anti‐steroidogenic roles by inhibiting growth and proliferation, and increasing apoptosis of GCs and TCs: miR‐145 attenuates mouse GC proliferation by targeting *Acvr1b* (Yan *et al.,* 2012), miR‐23a plays a pro‐apoptotic role in luteinized human GCs by reducing expression of *X‐linked inhibitor of apoptosis* (*XIAP*) and increasing *CAS3* (Yang *et al.,* 2012), and miR‐23a and miR‐27a also increase the apoptosis of human GCs by targeting *SMAD5* and positively regulating Fas‐induced death signals (Nie *et al.,* 2015). Expression of miR‐26b induces apoptosis of GCs in pigs by directly targeting *ATM*, a gene involved in DNA repair (Lin *et al.,* 2012), and by suppressing *SMAD4* signalling and *BCL2* (Liu *et al.,* 2014); let‐7 g promotes GC apoptosis in pigs by targeting *TGFBR1* and downregulating the TGFβ signalling pathway (Zhou *et al.,* 2015) as well as by targeting *CCND2* and *BCL2L1* and regulating MAPK and p53 signalling (Cao *et al.,* 2015). Additionally, a group of miRNAs including miR‐15a, and miR‐29a promote the accumulation of *BAX*, thus enhancing the apoptosis of human GCs (Sirotkin *et al.,* 2010). In addition, the follicular to luteal transition process and ovulation are stimulated by the presence of steroidogenic genes that are also regulated by miRNAs (Donadeu *et al.,* 2012; Iwamune *et al.,* 2014). Taken together, miRNAs control the biosynthesis of female steroidogenic hormones including oestrogen, progesterone, and androgens by regulating the proliferation, growth, and apoptosis of ovarian GCs and TCs.

## VII. miRNAs AS REGULATORS OF ZYGOTIC AND EARLY EMBRYO REPROGRAMMING

Endometrial miRNAs along with parental miRNAs from the germline or uterine fluid could participate in maternal–embryo crosstalk (Saadeldin *et al.,* 2015) and could play important roles during epigenetic reprogramming of the embryonic cells by regulating the expression and functions of genes related to early embryonic development (see Table S5).

### (1) Roles during fertilization, ZGA, and epigenetic reprogramming of preimplantation embryos

Recent data suggested that sperm‐borne miRNAs must be transferred into a fertilized embryo to maintain maternal and paternal communication. For example, Yuan *et al*. (2016) produced zygotes using *Drosha* conditional knockout (cKO) mouse‐derived miRNA‐depleted sperm and found the resulting zygotes to have reduced developmental potential. Of note, the developmental competence of these zygotes could be recovered through the injection of small miRNAs derived from WT sperm. Furthermore, they found 14 sperm‐borne miRNAs to be present in both WT sperm and the zygote, but not in oocytes, indicating that sperm‐borne miRNAs can affect the development potential of fertilized embryos. By contrast, most zygotic miRNAs are derived from maternal oocytes (Tang *et al.,* 2007). Altered miRNA expression in cumulus cells could result in a poor ovarian response in women undergoing *in vitro* fertilization (Karakaya *et al.,* 2015), and some miRNAs are highly specific to the blastocyst and are not expressed in the oocytes (Tulay *et al.,* 2015). In addition, sperm‐borne miR‐34c‐5p is thought to initiate the first cleavage divisions in the mouse (Liu *et al.,* 2012), and is positively correlated with intracytoplasmic sperm injection outcomes, as well as high‐quality embryos, implantation, pregnancy and live birth in humans (Cui *et al.,* 2015), and the presence of miR‐34c‐5p indicates viability and high‐quality spermatozoa in humans (Abu‐Halima *et al.,* 2014). Similarly, sperm‐borne miR‐34b/c and mir‐449a/b/c play a critical role in the *in vitro* first cleavage division of mouse embryos (Liu *et al.,* 2012; Yuan *et al.,* 2015). Based on the above findings, the presence of miR‐34c‐5p can be considered a non‐invasive biomarker for viable sperm and embryos in the mouse and human. In future, miR‐34c‐5p could be a universal biomarker for viable mammalian sperm and embryos; however, it requires intensive validation using other species.

Another study suggested that miR‐34b/c‐ and miR‐449‐null male mice were fertile, whereas miR‐34b/c and miR‐449 double‐knockout (miR‐dKO) male mice were infertile due to severe spermatogenic disruptions and oligoasthenoteratozoospermia (Yuan *et al.,* 2015). However, injection of miR‐dKO round spermatids into fertile mouse oocyte successfully gave rise to healthy offspring (Yuan *et al.,* 2015): indicating that this miRNA family is essential for normal spermatogenesis, but not for fertilization and embryonic development (Liu *et al.,* 2016). Sperm‐derived miR‐191, miR‐16, and miR‐146a are the most abundant miRNAs in early zygotes of mice (Yang *et al.,* 2016), but their roles are not clear. Despite being silenced functionally, the biogenesis of miRNAs is not abrogated during fertilization to ZGA in mice (Tang *et al.,* 2007; Suh *et al.,* 2010), suggesting that miRNAs might have important roles during transformation of zygotes to pluripotent blastocysts (Tang *et al.,* 2007; Suh *et al.,* 2010). Some miRNAs are stage‐specific: miR‐125a and miR‐496 are specific to four‐cell bovine embryos, while miR‐127, miR‐145, and miR‐496 are specific to eight‐cell bovine embryos. However, most miRNAs showed fluctuating expression patterns according to the stage of bovine embryonic development (Tesfaye *et al.,* 2009). This switch in miRNA expression patterns between ZGA and pluripotent blastocyst involves massive molecular rewiring (Hemberger, Dean, & Reik, 2009), and thus miRNAs might play a vital role during the transition of fertilized oocytes to pluripotent blastocysts (Svoboda & Flemr, 2010). Based on these findings, we can conclude that parental miRNAs might have important but limited roles during fertilization and the ZGA; however, miRNAs might be a key regulatory player during the developmental transition from the ZGA to pluripotent blastocyst.

**Figure 5:**
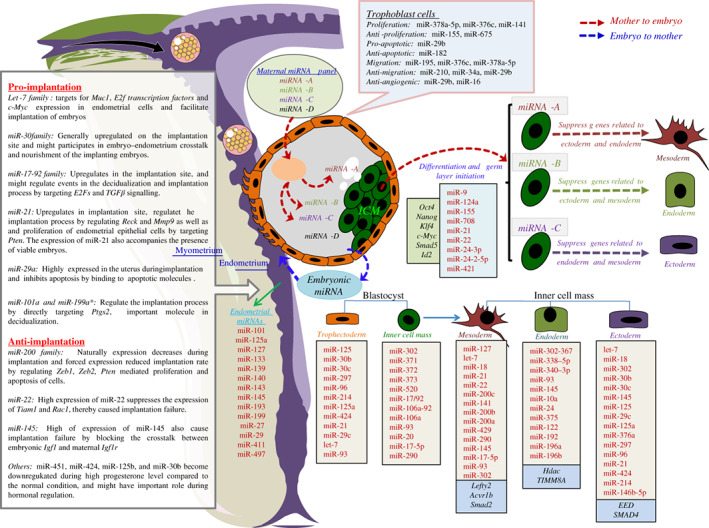
MicroRNA (miRNA) regulation during implantation and germ layer specification. Based on their functions, miRNAs are categorized as anti‐ or pro‐implantation miRNAs, pluripotency miRNAs, or miRNAs that promote differentiation and commitment of mesoderm, endoderm, ectoderm, and trophectoderm lineages. Parental miRNAs might be important regulators during embryonic germ layer specification; variation in the absorption of parental miRNAs could initiate variation in differentiation potential of the inner cell mass (ICM), and thus could direct the ICM towards different germ layers. For example, absorption of miRNA‐A by an ICM cell could suppress the endoderm and ectoderm lineage in that particular cell and thus facilitate mesoderm commitment. Similarly, the absorption of miRNA‐B by an ICM cell could suppress the mesoderm and ectoderm lineage in that respective cell and could direct the cell towards the endoderm lineage; the absorption of miRNA‐C by an ICM cell might facilitate ectoderm commitment by suppressing the mesoderm and endoderm lineages. Muc1, mucin 1, cell surface associated; c‐Myc, MYC proto‐oncogene, BHLH transcription factor; TGFβ, transforming growth factor beta; Reck, reversion inducing cysteine‐rich protein with kazal motifs; Mmp9, matrix metallopeptidase 9; Pten, phosphatase and tensin homolog; Ptgs2, prostaglandin‐endoperoxide synthase 2; Zeb1, zinc finger E‐box binding homeobox 1; Zeb2, zinc finger E‐box binding homeobox 2; Tiam1, T‐cell lymphoma invasion and metastasis 1; Rac1, Rac family small GTPase 1; Igf1, insulin like growth factor 1; Igf1r, insulin like growth factor 1 receptor; Oct4, octamer‐binding protein 4; Nanog, nanog homeobox; Klf4, kruppel like factor 4; Smad5, SMAD family member 5; Id2, inhibitor of DNA binding 2; Lefty2, left–right determination factor 2; Acvr1b, activin A receptor type 1B; Smad2, SMAD family member 2; Hdac, histone deacetylase; TIMM8A, translocase of inner mitochondrial membrane 8A; EED, embryonic ectoderm development; SMAD4, SMAD family member 4.

miR‐29b has been suggested to target *de novo* DNA methyltransferases (*Dnmt3a* and *Dnmt3b*) transcripts in PGCs (Takada *et al.,* 2009). The suppression of *Dnmt* by miRNAs safeguards proper DNA demethylation and establishes the new imprinting of genes. Similarly, *Dnmt3a*/*3b* expression was negatively regulated by miR‐29b at the time of mouse ZGA (Zhang *et al.,* 2015). Given that DNA methylation plays a critical role in the morula‐to‐blastocyst transition, these data suggest a feedback relationship between the regulation of miRNA expression and DNA methylation. In addition, pluripotency markers including *kruppel like factor 4* (*Klf4*) and *nanog homeobox* (*Nanog*) were downregulated in the morula stage upon miR‐29b inhibition (Wang *et al.,* 2016), indicating the involvement of miR‐29b in the regulation of pluripotency. Similarly, the miR‐290/302 cluster is involved with embryonic pluripotency, although an opposing role of this cluster was shown in one study (Gu *et al.,* 2016). Again, differential expression of miRNAs subsets during trophectoderm specification (Viswanathan *et al.,* 2009) or severe preeclamptic placentas (Pineles *et al.,* 2007) evidence a role for miRNAs during the implantation process. The endometrial miRNAs along with miRNAs from the uterine fluid also could participate during maternal–embryo crosstalk and play an important role during the epigenetic reprogramming of the embryonic cells (Fig. 5). Thus, accumulating evidence suggests that miRNAs are at least indirectly engaged in the epigenetic reprogramming process of early embryos.

### (2) miRNAs regulate germ‐layer specification in early embryos

During the gastrulation stage, embryonic stem cells (ESCs) differentiate to epiblast stem cells (EpSCs) and then EpSCs begin to differentiate into the endoderm, mesoderm and ectoderm lineages. Differentiation starts with the downregulation of pluripotency markers including *octamer‐binding protein 4* (*Oct4*), *Nanog*, *Klf4*, and *c‐Myc*, which are regulated by miR‐24‐3p and miR‐24‐2‐5p, while expression of *protein arginine methyltransferase 7* (*Prmt7*) inhibits miR‐24‐3p and miR‐24‐2‐5p. Thus, miR‐24‐3p and miR‐24‐2‐5p create a feedback loop with *Prmt7* and regulate the differentiation of mouse ESCs (Lee *et al.,* 2016). Furthermore, miR‐124a in humans, and miR‐200a/b/c, miR‐141 and miR‐429 in the mouse regulate early gastrulation‐stage embryos (Berardi *et al.,* 2012). In ESCs, miR‐191 and miR‐16‐1 are abundant, and inhibit the formation of mesoderm by downregulating *Smad2*, an essential component of *Activin–Nodal* signalling (Hadjimichael *et al.,* 2016), while miR‐23a may suppress the differentiation of ESCs towards the endoderm and ectoderm lineages (Hadjimichael *et al.,* 2016). By contrast, expression of miR‐9, miR‐124a, miR‐155, and miR‐708 facilitate the differentiation of mouse ESCs (Marson *et al.,* 2008), and the overexpression of miR‐21 facilitates the commitment of mesodermal tissues such as adipose and bone (Eguchi *et al.,* 2013; Kang *et al.,* 2013). Similarly, miR‐303‐367 and miR‐290‐295 clusters may play an important role during the patterning and specification of embryonic germ layers in humans and mice, respectively, by regulating *Nodal* signalling (Vidigal & Ventura, 2012). During this stage, BMPs, Activin/Nodal, and canonical Wnts signals play critical roles for the transition of inner cell mass (ICM) to mesoderm or endoderm, whereas non‐canonical Wnts, fibroblast growth factors (FGFs), Notch, and hedgehog (Hh) signalling are closely involved in the transition of *mesoderm posterior 1 homolog (Mesp1)*‐specified mesoderm to cardiac mesoderm.

Embryonic mesoderm shows predominant expression of miR‐145 and miR‐302 in humans and miR‐290, miR‐93, and miR‐17‐5p in mice (Berardi *et al.,* 2012). Similarly, the miR‐200 family is known for its role in early mesoderm commitment by regulating s*nail family transcriptional repressor 1* (*Snail*) (Gill *et al.,* 2011). miR‐145 accelerates mesoderm and ectoderm commitment by targeting *OCT4*, *sex determining region Y‐box 2* (*SOX2*), and *KLF4* in human ESCs (Xu *et al.,* 2009), and miR‐127 induces differentiation of mouse ESCs to the mesendoderm lineage by targeting *left–right determination factor 2* (*Lefty2*) and regulating *Nodal* signalling (Ma *et al.,* 2016b). let‐7 and miR‐18 target *Acvr1b* and *Smad2*, respectively, and may attenuate Nodal responsiveness as well as bias the blastomeres towards the ectoderm and mesoderm (Colas *et al.,* 2012). Thus, *in vivo* blocking of let‐7 and miR‐18 activity may divert the presumptive mesoderm and ectoderm towards the endoderm (Colas *et al.,* 2012).

Embryonic endoderm expresses miR‐145, miR‐10a, miR‐24, miR‐375, miR‐122, miR‐192, miR‐196a, and miR‐196b in humans and miR‐93, miR‐338‐5p, and miR‐340‐3p in mice (Berardi *et al.,* 2012). miR‐421 represses *Oct4* and *BMP* signalling components such as *Smad5* and *inhibitor of DNA binding 2* (*Id2*) in mouse ESCs (Hadjimichael *et al.,* 2016), while miR‐338‐5p and miR‐340‐3p promote endodermal differentiation of mouse ESCs by regulating *histone deacetylase* (*Hdac*) activity (Fu *et al.,* 2011), and miR‐375 plays important roles in endoderm commitment in human ESCs by targeting *translocase of inner mitochondrial membrane 8A* (*TIMM8A*) (Hinton *et al.,* 2010).

Embryonic ectoderm also represents a unique miRNA profile, and predominant expression of miR‐125, miR‐30b, and miR‐30c have been observed in embryonic human ectoderm, while miR‐29c, miR‐125a, miR‐376a, miR‐297, miR‐96, miR‐21, let‐7, miR‐424, and miR‐214 were predominant in the embryonic ectoderm of mice (Berardi *et al.,* 2012). In addition, miR‐30b modulates formation of early human ectoderm by regulating *embryonic ectoderm development* (*EED*) (Song *et al.,* 2011), miR‐125 and miR‐146b‐5p regulate the commitment of the neural lineage in humans by targeting *SMAD4* (Boissart *et al.,* 2012; Zhang *et al.,* 2017), miR‐17‐5p promotes proliferation and inhibits the differentiation of lung epithelial progenitor cells in mice by targeting *RB transcriptional corepressor like 2* (*Rbl2*) (Lu *et al.,* 2007), and overexpression of miR‐125b‐2 represses the differentiation of mouse ESCs to the ectodermal lineage by regulating *Lin28* and *differentiation of embryonic stem cells 1* (*Dies1*) (Deng *et al.,* 2015).

miR‐125, miR‐30b, and miR‐30c are specific to human embryonic trophectoderm, while the expression of miR‐297, miR‐214, miR‐96, miR‐125a, miR‐21, miR‐93, miR‐424, miR‐29c, let‐7, and miR‐376a was detected in mouse trophectoderm (Berardi *et al.,* 2012). Additionally, mouse blastocysts showed upregulated expression of a panel of miRNAs and downregulated expression of miR‐376a compared to the morula stage, indicating the involvement of related miRNAs during trophectoderm commitment (Viswanathan *et al.,* 2009).

Finally, early embryos may be enriched with a panel of parental miRNAs (Fig. 5), either through gametes (Tang *et al.,* 2007; Yuan *et al.,* 2016) or embryo–maternal crosstalk (Saadeldin *et al.,* 2015; Bidarimath *et al.,* 2017). Exosomes and cell‐membrane‐derived micro‐ and nano‐vesicles could be the main medium for this embryo–maternal crosstalk (Diehl *et al.,* 2012; Saadeldin *et al.,* 2015), however, miRNAs could also be carried through the circulatory system by apoptotic bodies, RNA‐binding lipoproteins and Argonaute protein complex (Ling, Bao‐feng, & Jing, 2013; Sohel, 2016). These circulating miRNAs originating from one cell or organ could be transported to and absorbed by neighbouring or distant cells, for example, miRNAs originating from tumour cells could be absorbed by other healthy cells leading to their transformation to malignancy (Guo *et al.,* 2017). Thus, it could be assumed that each cell of the embryonic ICM randomly absorbs different maternal miRNAs delivered through the circulatory system; this variation in miRNA intake among the cells of the embryonic ICM may lead the cells towards different lineages. For example, one cell may absorb a miRNA‐A panel, leading to the mesoderm lineage, another may absorb a miRNA‐B panel and commit to the endoderm lineage, while a third cell may absorb a miRNA‐C panel to specify the ectoderm lineages (Fig. 5). However, further in‐depth experiments, both *in vitro* and *in vivo*, are required to support these hypotheses.

### (3) miRNAs regulate pregnancy by mediating adhesion molecules, vasculature and angiogenesis

miRNAs involved in the implantation process can be categorized into two groups, anti‐ or pro‐implantation miRNAs (Fig. 5); these miRNAs are potent regulators of many genes related to adhesion, angiogenesis, and vasculature (see Table S5), as well as foetal–maternal immune tolerance following endometrial reception of the conceptus (Bidarimath *et al.,* 2014, 2015). Alterations in expression of these miRNAs in the uterine endometrium could cause implantation failure as well as disease (Hiroki *et al.,* 2010). For example, the let‐7 family regulates the expression of *mucin 1*, *cell surface associated* (*Muc1*) and facilitates implantation of mouse embryos (Inyawilert *et al.,* 2015), the miR‐30 family is generally upregulated at the implantation site and may participate in embryo–endometrium crosstalk and nourishment of the implanting embryo (Moreno‐Moya *et al.,* 2014), the miR‐17‐92 cluster is upregulated at the implantation site, and may regulate the decidualization (morphological and functional changes of the endometrium in preparation for pregnancy) and implantation process by targeting *E2F* and *TGFβ* signalling (Mogilyansky & Rigoutsos, 2013). Similarly, miR‐21 is upregulated at the implantation site and regulates *matrix metallopeptidase 2* (*Mmp2*) and *matrix metallopeptidase 9* (*Mmp9*) expression by targeting *reversion inducing cysteine‐rich protein with kazal motifs* (*Reck*) during implantation in mice (Hu *et al.,* 2008; Carletti & Christenson, 2009) and *PTEN* during the proliferation of endometrial epithelial cells (Qin *et al.,* 2012). miR‐29a is highly expressed in the uterus during implantation and inhibits apoptotic molecules (Xia *et al.,* 2014), and miR‐101a and miR‐199a* regulate the implantation process by directly targeting *Cox‐2* (*Ptgs*), an important molecule for decidualization (Chakrabarty *et al.,* 2007). A panel of miRNAs including *miR‐10a*, miR‐*27a*, miR‐*29c*, miR‐*323*, miR‐*331‐5p*, miR‐*339‐3p*, miR‐*374‐5p*, and miR‐*935* are reported to be associated with spontaneous foetal arrest in pigs (Wessels *et al.,* 2013), while miR‐296‐5P, miR‐150, miR‐17P‐5P, miR‐18a, and miR‐19 are associated with angiogenesis of the endometrial tissues in pigs (Bidarimath *et al.,* 2015), and are differentially expressed between healthy and arresting conceptus attachment site (Bidarimath *et al.,* 2015). Thus these miRNAs could be used to assess foetal health, although this requires validation with other mammalian species. Additionally, some miRNAs participate in the implantation process indirectly, such as by promoting the proliferation of trophoblast cells (Luo *et al.,* 2012; Fu *et al.,* 2013), by facilitating trophoblast migration (Bai *et al.,* 2012; Luo *et al.,* 2012; Fu *et al.,* 2013), by inhibiting the apoptosis of trophoblasts (Pineles *et al.,* 2007), and by participating in conceptus–endometrial crosstalk (Bidarimath *et al.,* 2017).

By contrast, the expression of oestradiol‐ and progesterone‐regulatory miR‐200 family members decreases naturally, while its targets *Zeb1* and *zinc finger E‐box binding homeobox 2* (*Zeb2*) are upregulated during implantation in mice (Jimenez *et al.,* 2016), and forced expression of miR‐200a reduced the implantation rate of mouse embryos by regulating *Pten*‐mediated proliferation and apoptosis of cells (Shen *et al.,* 2013). Overexpression of miR‐22 suppresses *T‐cell lymphoma invasion and metastasis 1* (*Tiam1*) and *Rac family small GTPase 1* (*Rac1*), causing implantation failure (Ma *et al.,* 2015), while overexpression of miR‐145 also causes implantation failure in mice by blocking crosstalk between embryonic *Igf1* and maternal *insulin like growth factor 1 receptor* (*Igf1r*) (Kang *et al.,* 2015). Similarly, miR‐451, miR‐424, miR‐125b, and miR‐30b are downregulated when progesterone is elevated compared to that under normal conditions (Li *et al.,* 2011). Oestrogen‐mediated inhibition of miR‐705 facilitates *Mmp9* translation (Carletti & Christenson, 2009), which may be important for proper implantation of embryos and thus pregnancy. Some other miRNAs play anti‐implantation roles by inhibiting proliferation (Dai *et al.,* 2012; Gao *et al.,* 2012), inducing apoptosis (Li *et al.,* 2013), and inhibiting migration (Zhang *et al.,* 2012; Umemura *et al.,* 2013) of the trophoblasts, as well as by suppressing vascularization and angiogenesis during implantation of embryos (Wang *et al.,* 2012).

Active or pseudopregnancy could be confirmed by the expression of miR‐21, which is expressed only in the presence of active embryos (Hu *et al.,* 2008; Luense *et al.,* 2009). As discussed earlier, miRNAs also participate in the maintenance of pregnancy by regulating angiogenesis and vasculogenesis of luteal tissues. In brief, miR‐125b, miR‐145, mir‐31, miR‐503 and miR‐21 regulate interferon‐mediated cell death, and vascularization and angiogenesis in ruminant CL (Donadeu *et al.,* 2012), and miR‐378 regulates the maintenance of the bovine CL by regulating its target *IFNGR1* (Ma *et al.,* 2011). miR‐17‐5p and let‐7 play important roles in CL formation by targeting the anti‐angiogenic gene *Timp1* (Otsuka *et al.,* 2008). In sheep, miR‐199a‐3p, miR‐145 and miR‐503 are expressed differentially at different stages of the CL and are involved in fibrosis, and immune and inflammatory responses (Donadeu *et al.,* 2012). Taken together, miRNAs mediate implantation and pregnancy in mammals by regulating hormone levels throughout pregnancy, maintaining crosstalk between the embryo and mother, and facilitating angiogenesis and vascularization of the endometrium and luteal tissues.

## VIII. CONCLUSIONS

(1) Loss of miRNA processing machinery could impair reproduction at the molecular level and causes infertility or sterility to different extents depending on sex, age and stage of loss, but the external sex characteristics along with mating behaviour remain normal in both males and females.

(2) miRNAs are mostly indispensable during pre‐ and post‐fertilization stages (such as sex differentiation, germline establishment, gametogenesis, preimplantation development and implantation of embryos) but might have very limited roles during the fertilization event. miRNA‐loss‐mediated infertility therefore derives from the malformation of gametes, not because of its absence during the fertilization event itself.

(3) miRNAs could have a direct involvement in the global silencing of X‐linked gene expression during the MSCI process of spermatogenesis and in the initiation of ZGA and the first embryonic cell division.

(4) Steroidogenesis in both sexes is regulated by potent feedback loops comprising miRNAs, genes, and hormones; deregulation in any of which could impair the whole process of mammalian reproduction.

(5) miRNAs could be used as fertility biomarkers for both gametes, and also to differentiate between pseudo and viable pregnancy.

(6) Parental miRNAs (derived from gametes or embryo–maternal crosstalk) could play very important roles in epigenetic modifications and germ‐layer specification of embryos. They may also be important in the implantation and maintenance of pregnancy by regulating angiogenesis and vascularization of the endometrium tissues and CL.

(7) Much work remains to be done to understand the roles of miRNAs in mammalian reproduction, especially in MSCI, the first embryonic cell division and ZGA, germ‐layer specification and embryo–maternal crosstalk.

## Supporting information


**Table S1.** Functions of microRNAs (miRNAs) in sex specification and commitment of the mammalian reproductive system.
**Table S2.** Functions of microRNAs (miRNAs) in spermatogenesis and spermiogenesis.
**Table S3.** Functions of microRNAs (miRNAs) during folliculogenesis and oogenesis.
**Table S4.** Functions of microRNAs (miRNAs) in the regulation of steroidogenic cells and hormonal balance.
**Table S5.** Functions of microRNAs (miRNAs) in fertilization, implantation and germ‐layer specification.
