# Supplementary material for: Roles of microRNAs in mammalian reproduction: from the commitment of germ cells to peri‐implantation embryos
Source: Biol Rev Camb Philos Soc. 2018 Aug 27;94(2):415–38. doi: 10.1111/brv.12459 (PMC7379200; doi:10.1111/brv.12459)
Supplement: Supplementary file 1 — Table S1. Functions of microRNAs (miRNAs) in sex specification and commitment of the mammalian reproductive system. Table S2. Functions of microRNAs (miRNAs) in spermatogenesis and spermiogenesis. Table S3. Functions of microRNAs (miRNAs) during folliculogenesis and oogenesis. Table S4. Functions of microRNAs (miRNAs) in the regulation of steroidogenic cells and hormonal balance. Table S5. Functions of microRNAs (miRNAs) in fertilization, implantation and germ‐layer specification. [file BRV-94-415-s001.docx]

**Table S1.** Functions of microRNAs (miRNAs) in sex specification and commitment of the mammalian reproductive system. PGCs, primordial germ cells.

| **miRNA** | **Target genes** | **Predominant expression site/role** | **Species** | **Reference** |  |
| --- | --- | --- | --- | --- | --- |
| miR-17-92 and miR-290-295 |  | Migration and colonization of PGCs | Mouse | [Hayashi *et al.* (2008](#_ENREF_73)); [Medeiros *et al.* (2011](#_ENREF_136)) |  |
| let-7 family (let-7a, let-7d, let-7e, let-7f and let-7g), miR-125a and miR-9 | *Blimp1* | *Hox* gene upregulation and male germline commitment | Mouse | [Ohinata *et al.* (2005](#_ENREF_154)); [Robertson *et al.* (2007](#_ENREF_168)); [Vincent *et al.* (2005](#_ENREF_215)); [West *et al.* (2009](#_ENREF_222)) |  |
| let-7, miR-23b, miR-21 |  | Differentiation of PGCs | Mouse | [Brieno-Enriquez *et al.* (2015](#_ENREF_21)) |  |
| miR-21a-5p, miR-17-5p, miR-20a-5p, miR-18a-5p, and miR-19b-3p | *Pten* | Maintain pluripotency of male PGCs | Mouse | [Garcia-Lopez *et al.* (2015](#_ENREF_60)) |  |
| miR-93-5p, miR-20a-5p, and miR-17-5p | *Stat* | Maintain pluripotency of male PGCs | Mouse | [Garcia-Lopez *et al.* (2015](#_ENREF_60)) |  |
| miR-290-295 cluster (miR-293, miR-291a-5p, miR-290-5p, and miR-294*) |  | Maintain pluripotency of male PGCs | Mouse | [McIver *et al.* (2012*b*](#_ENREF_135)); [Zheng *et al.* (2011](#_ENREF_260)) |  |
| miR-221, miR-222 | *Kit* | Sustain pluripotent status and reproducing ability of male GSCs | Mouse | Q.E. [Yang *et al.* (2013](#_ENREF_240)) |  |
| miR-22 | *ESR1* | Enhance male specification and suppress female specification | Sheep | [Torley *et al.* (2011](#_ENREF_203)) |  |
| miR-202-5p, miR-202-3p, miR-140-3p, miR-140-5p |  | Early testes and Leydig cells development | Mouse | [Eggers *et al.* (2014](#_ENREF_53)) |  |
| miR-741, miR-742, miR-743, miR-465-3p, miR-463, miR-465-5p, miR-470, miR-471 | |  | Testes | Mouse | [Watanabe *et al.* (2006](#_ENREF_220)) |
| miR-184, miR-202-5p, miR-508-3p, miR-509-5p | |  | Testes | Human | Q. [Yang *et al.* (2013](#_ENREF_237)) |
| miR-449a | |  | Testes | Human, mouse | Z. [Luo *et al.* (2015](#_ENREF_124)) |
| let-7, miR-923, miR-202, miR-21, miR-145 | |  | Testes | Pig | [Luo *et al.* (2010](#_ENREF_121)) |
| miR-449, miR-34b, miR-34c, miR-181d, miR-214, miR-122a, miR-16, miR-101 | | *AQN-1*, *HAS3*, *RNF4*, *SMCP*, *SPAM1* | Differentially expressed between mature and immature testes | Pig | [Luo *et al* (2010).](#_ENREF_121) |
| miR-485, miR-878-5p | |  | Sex specification | Mouse | [Rakoczy *et al.* (2013](#_ENREF_161)) |
| miR-140-5p and miR-140-3p | |  | Leydig cells development | Mouse | [Rakoczy *et al.* (2013](#_ENREF_161)) |
| miR-29a | | *Nasp* | Inhibits epididymal cell proliferation | Rat | [Ma *et al.* (2012](#_ENREF_129)) |
| let-7c-5p, let-7b-5p, miR-375-3p, miR-9-5p, miR-467d-3p, and miR-200c-3p | |  | Caput of epididymis | Mouse | [Nixon *et al.* (2015*a*](#_ENREF_149)) |
| miR-410-3p, miR-486-5p, and miR470c-5p | |  | Cauda of epididymis | Mouse | [Nixon *et al.* (2015*a*](#_ENREF_149)) |
| miR-890, miR-892a, miR-892b, miR-891a and miR-891b, miR-200b, miR-10a, miR-424, miR-542, miR-31, miR-183 and miR-363 | |  | Differentially expressed in different segments of epididymis | Human | [Belleannee *et al.* (2012](#_ENREF_10)) |
| miR-7b-5p, miR-9-5p, miR-31-5p, miR-92a-3p, miR-106-5p, miR-126-3p, miR-150-5p, miR-204-5p, miR-222-3p, miR-322-5p | |  | Differentially expressed in different segments of epididymis | Mouse | [Nixon *et al.* (2015*a*](#_ENREF_149)) |
| miR-145 | | *CLDN10* | Regulates blood–epididymis barrier | Human | [Belleannee *et al.* (2012](#_ENREF_10)) |
| miR-200 family | | *Bcap29*, *Rab21*, *Slc23a1*, *Dek* | Predominant in caput compared to cauda of the epididymis | Rat | [Chu *et al.* (2015](#_ENREF_33)) |
| miR-149-5p, miR-148a-3p, miR-28-3p, miR-196b-5p, miR-151-3p | |  | Spermatozoal miRNAs derived from the epididymis | Mouse | [Nixon *et al.* (2015*b*](#_ENREF_150)) |
| miR-29b | | *Dnmt3a*, *Dnmt3b* | Female germline and gonad commitment | Mouse | [Takada *et al.* (2009](#_ENREF_196)) |
| miR-124 | | *Sox9* | Architecture of ovary | Mouse | [Eggers *et al.* (2014](#_ENREF_50)) |
| let-7 family, miR-21, miR-99a, miR-125b, miR-126, miR-143, miR-145, miR-199b, miR-25, miR-106a | |  | Ovary | All species | [Li *et al.* (2015](#_ENREF_106)) |
| miR-26a, miR-125b, let-7c | |  | Ovary | Human, mouse | [Ahn *et al.* (2010](#_ENREF_4)); [Liang *et al.* (2007](#_ENREF_108)); [Nothnick (2012](#_ENREF_151)) |
| miR-709 | |  | Ovary | Mouse | [Choi *et al.* (2007](#_ENREF_32)) |
| miR-133b | | *TAGLN2* | Ovary | Human, mouse | [Xiao *et al.* (2014](#_ENREF_228)) |
| miR-10b, miR-26a, miR-21, miR-140, miR-101 | |  | High in the ovary compared to testes | Pig | [Li *et al.* (2015](#_ENREF_106)) |
| miR-378, miR-1, miR-206, miR-379, miR-127, miR-411 | |  | Low in the ovary compared to testes | Pig | [Li *et al.* (2015](#_ENREF_106)) |
| miR-26a, miR-26b, miR-21 | |  | Fallopian tubes | Human | [Nothnick (2012](#_ENREF_151)) |
| miR-26a, miR-26b, miR-125b, miR-16, let7c | |  | Uterus | Human | [Nothnick (2012](#_ENREF_151)) |
| miR-26a, miR-26b, miR-29a, miR-21, miR-23b, miR-24, miR-99a | |  | Cervix | Human | [Nothnick (2012](#_ENREF_151)) |
| let-7, miR-503, miR-672, miR-465, miR-21-5p, miR-143-3p | |  | Housekeeping miRNAs in both sexes |  | [Ahn *et al.* (2010](#_ENREF_4)); [Li *et al.* (2015](#_ENREF_106)) |
| miR-34 family | |  | Fertility marker for both gametes | Cattle | [Tscherner *et al.* (2014](#_ENREF_205)) |

**Table S2.** Functions of microRNAs (miRNAs) in spermatogenesis and spermiogenesis. SSCs, spermatogonial stem cells; GSCs, germline stem cells; TCA, tricarboxylic acid cycle; MSCI, meiotic sex chromosome inactivation; GC-1 SPG, spermatogonia cell line.

| **miRNA** | **Target genes** | **Function** | **Species** | **Reference** |
| --- | --- | --- | --- | --- |
| miR-15a | *Ccnt2* | Early specification of male germline | Mouse | [Teng *et al.* (2011](#_ENREF_198)) |
| miR-302 family (miR-302a-3p/-5p, miR-302d-3p and miR-302c-3p), miR-7662-5p and miR-421-5p | *Cdkn1a* | Specific to male germline cells | Mouse | [Garcia-Lopez *et al.* (2015](#_ENREF_60)) |
| miR-143-3p |  | Regulator of proliferation and differentiation of male germ cells | Mouse | [Garcia-Lopez *et al.* (2015](#_ENREF_60)) |
| miR-3102-5p.2-5p and miR-1188-5p |  | Expressed in differentiated spermatogonia | Mouse | [Garcia-Lopez *et al.* (2015](#_ENREF_60)) |
| miR-293 |  | Differentially expressed between gonocytes and spermatogonia | Mouse | [McIver *et al.* (2012*b*](#_ENREF_135)) |
| miR-21, miR-34c, miR-182, miR-183, and miR-146a |  | Differentially expressed between Thy1- and Thy1+ SSCs | Mouse | [Niu *et al.* (2011](#_ENREF_148)) |
| miR-221, miR-222 | *c-Kit* | Maintain the stemness of SSCs | Mouse | Q.E. [Yang *et al.* (2013](#_ENREF_240)) |
| miR-20 and miR-106a | *Stat3*, *Ccnd1* | Promote self-renewal of SSCs by increasing *Pcna*, *Plzf* and reducing *Kit* | Mouse | [He *et al.* (2013](#_ENREF_74)) |
| miR-290 and miR-302 |  | Maintaining the pluripotency of GSCs | Mouse | [Zovoilis *et al.* (2008](#_ENREF_263)) |
| miR-184 | *Ncor2* | Germline cell proliferation and enrichment | Mouse | [Wu *et al.* (2011](#_ENREF_224)) |
| Let-7 family |  | Spermatogonial differentiation | Mouse | [Tong *et al.* (2011](#_ENREF_201)) |
| miR-17-92 and miR-106b-25 cluster |  | Spermatogonial differentiation | Mouse | [Tong *et al.* (2012](#_ENREF_202)) |
| miR-146 | *Med1*,  *c-Kit*, *Soh1h2* | Modulates differentiation of SSCs | Mouse | [Huszar & Payne (2013](#_ENREF_84)) |
| miR-34c | *Nanos2* | Differentiation of SSCs | Mouse | [Yu *et al.* (2014](#_ENREF_250)) |
| miR-449 | *NOTCH1*, *BCL2* | Differentiation or survival of germline cells | Monkey, human | [Yan *et al.* (2009](#_ENREF_235)) |
| miR-34b, miR-34c | *NOTCH1*, *NOTCH2*, *TGIF2*, *CDK4*, *MYC* | Differentiation or survival of germline cells | Monkey, human | [Bouhallier *et al.* (2010](#_ENREF_20)); [Yan *et al.* (2009](#_ENREF_235)) |
| Let-7 | *Hmga2* | Structural organization and differentiation of germ cells | Mouse | [Chieffi *et al.* (2002](#_ENREF_31)); [Curry *et al.* (2011](#_ENREF_38)) |
| miR-22 | *ODF1* | Structural organization and differentiation of germ cells | Human | [Curry *et al.* (2011](#_ENREF_38)); [Zhao *et al.* (2007](#_ENREF_259)) |
| miR-15b | *GDI1*, *IDH3A* | Regulates TCA-cycle-mediated energy metabolism | Pig | [Curry *et al.* (2011](#_ENREF_38)) |
| Let-7 family | *Igf1* | Regulates spermatogonial differentiation | Mouse | [Shen *et al.* (2014](#_ENREF_178)) |
| miR-383 | *IRF1* | Regulates cell cycle molecules and cell division | Human | [Lian *et al.* (2010](#_ENREF_107)); [Luo *et al.* (2016](#_ENREF_122)) |
| miR-294 and miR-295 |  | Specific to SSCs | Mouse | [Smorag *et al.* (2012](#_ENREF_183)) |
| miR-221, miR-203 | *c-Kit*, *Rbm44* | SSC maintenance | Mouse | [Smorag *et al.* (2012](#_ENREF_183)) |
| miR-34b-5p | *Cdk6* | Meiotic differentiation | Mouse | [Smorag *et al.* (2012](#_ENREF_183)) |
| miR-184, miR-1225-5p and miR-30c-2-3p |  | Downregulated in pachytene spermatocytes compared to spermatogonia | Human | [Liu *et al.* (2015](#_ENREF_116)) |
| miR-126-3p, let-7a-5p and miR-125b-5p |  | Upregulated in pachytene spermatocytes compared to spermatogonia | Human | [Liu *et al.* (2015](#_ENREF_116)) |
| miR-184 | *E2F1* | Play role during spermatogenesis | Human | [Liu *et al.* (2015](#_ENREF_116)) |
| miR-1225-5p | *ETV1* | Play role during spermatogenesis | Human | [Liu *et al.* (2015](#_ENREF_116)) |
| miR-30c-2-3p | *TNFAIP8l2* | Play role during spermatogenesis | Human | [Liu *et al.* (2015](#_ENREF_116)) |
| miR-126-3p | *TOM1* | Play role during spermatogenesis | Human | [Liu *et al.* (2015](#_ENREF_116)) |
| let-7a-5p | *TGFBR1* | Play role during spermatogenesis | Human | [Liu *et al.* (2015](#_ENREF_116)) |
| miR-125b-5p | *BMPR2* | Play role during spermatogenesis | Human | [Liu *et al.* (2015](#_ENREF_116)) |
| miR-100-5p, miR-34c-5p, miR-34b-5p and miR-206 |  | Differentially expressed between pachytene spermatocytes and round spermatids | Human | [Liu *et al.* (2015](#_ENREF_116)) |
| miR-21, miR-140-3p, miR-103, miR-30a, miR-101b and miR-99b |  | Regulates B-spermatogonia to primary spermatocyte transformation | Mouse | M. [Luo *et al.* (2015](#_ENREF_123)) |
| miR-24 | *MBD6*, *H2AX* | Regulates meiosis division during spermatogenesis | Hamster | [Marcon *et al.* (2008](#_ENREF_131)); [McIver *et al.* (2012*a*](#_ENREF_134)) |
| miR-355, miR-181b, and miR-181c | *Rsbn1* | Transcriptional regulation of haploid germ cells | Mouse | [McIver *et al.* (2012*a*](#_ENREF_134)); [Yan *et al.* (2007](#_ENREF_236)) |
| miR-18 | *Hsf2* | Regulates male germ cells maturation | Mouse | [Bjork *et al.* (2010](#_ENREF_17)) |
| miR-136, miR-743a, and miR-463* |  | Promote differentiation of spermatogonia to mature germ cells |  | [Golestaneh *et al.* (2009](#_ENREF_64)) |
| mir-718-3p, mir-883a-3p and mir-883a-5p |  | Putative regulator of MSCI process | Mouse | [Song *et al.* (2009](#_ENREF_188)) |
| miR-18, miR-183, miR-16 | *Atm* | Involved in the maintenance of chromosomal stability | Mouse | [Modzelewski *et al.* (2015](#_ENREF_140)) |
| miR-762 | *γ-H2AX* | Involved in the maintenance of chromosomal stability | Pig | C. [Ma *et al.* (2016](#_ENREF_125)) |
| miR-122 | *Tnp2* | Regulates substitutes of histones during spermatogenesis | Mouse | [Yu *et al.* (2005](#_ENREF_251)) |
| miR-469 | *Tnp2*, *Prm2* | Chromatin compaction and shaping of the sperm head | Mouse | [[de Mateo & Sassone-Corsi](#_ENREF_46) (2014](#_ENREF_46)) |
| miR-3473 and miR-221 |  | Predominant in germline cells and zygotes | Mouse | [Garcia-Lopez *et al.* (2015](#_ENREF_60)) |
| miR-10b-5p, miR-10a-5p, miR-143-3p, miR-141-3p and miR-30a-5p |  | Derived from the epididymis and enrich spermatozoa during epididymal storage | Mouse | [Reilly *et al.* (2016](#_ENREF_162)) |
| miR-891b, miR-892b, miR-892a, miR-888 and miR-890 |  | Regulate sperm motility | Human | [Qing *et al.* (2017](#_ENREF_160)) |
| let-7a, let-7d, let-7e, miR-26a, miR-98, miR-181a, miR-505, and miR-676 |  | Downregulated in ejaculated sperm compared to epididymal sperm | Pig | [Chang *et al.* (2016](#_ENREF_26)) |
| miR-19b, miR-34c, miR-92a, miR-105-1, miR-224, miR-363, and miR-504 |  | Upregulated in ejaculated sperm compared to epididymal sperm | Pig | [Chang *et al.* (2016](#_ENREF_26)) |
| miR-16 and miR-34 |  | Involved in the senescence and apoptosis of sperm | Pig | Z. [Luo *et al.* (2015](#_ENREF_124)) |
| miR-29a, miR-29b, and miR-29c | *Dnmt1*, *Dnmt3a*, *Dnmt3b*, *Mcl-1* | Putative regulator of germ cells apoptosis | Rat | [Meunier *et al.* (2012](#_ENREF_139)) |
| miR-122 | *Bcl-w*, *Ccng1* | Putative regulator of anti-apoptotic genes | Mouse | R. [Chen *et al.* (2015](#_ENREF_28)) |
| miR-16 | *Ccnd1* | Promotes apoptosis of GC-1 SPG cells | Mouse | [Li *et al.* (2016*b*](#_ENREF_103)) |
| miR-17-92 cluster | *E2F1* | Protects spermatozoa by playing an anti-apoptotic role | Human | [Novotny *et al.* (2007](#_ENREF_152)) |
| 17-92 cluster (miR-17, miR-18a, miR-19a, miR-20a, miR-19b-1, and miR-92a) | *Bim*, *Stat3*,  *c-Kit*, *Socs3* | Regulates germ cell proliferation, differentiation and apoptosis | Mouse | [Xie *et al.* (2016](#_ENREF_229)) |

**Table S3.** Functions of microRNAs (miRNAs) during folliculogenesis and oogenesis. GV, germinal vesical; DF, dominant follicle; SF, subordinate follicle; GC, granulosa cell; GnRH, gonadotropin-releasing hormone; LH, luteinizing hormone; FSH, follicle-stimulating hormone; CL, corpus luteum.

| **miRNA** | **Target genes** | **Function** | **Species** | **Reference** |
| --- | --- | --- | --- | --- |
| miR-125b | *LIF* | Regulates follicle development | Cattle | [Donadeu *et al.* (2012](#_ENREF_52)) |
| miR-199a-3p | *PTGS2* | Regulates follicle development | Cattle | [Donadeu *et al.* (2012](#_ENREF_52)) |
| miR-145 | *CDKN1A* | Regulates follicle development | Cattle | [Donadeu *et al.* (2012](#_ENREF_52)) |
| miR-145 | *Tgfbr2*, *Acvr1b*, *Smad3*, *Smad5* | Development and maintenance of primordial follicles | Mouse | S. [Yang *et al.* (2013](#_ENREF_241)) |
| miR-376a | *Pcna* | Regulates primordial follicle assembly | Mouse | [Zhang *et al.* (2014](#_ENREF_254)) |
| miR-143 | *Cdks*, *Ccnb1*, *Ccnd2*, *Ccne2* | Inhibits primordial follicle formation | Mouse | Q. [Zhang *et al.* (2013](#_ENREF_257)) |
| miR-224 | *Smad4* | Enhances folliculogenesis by inducing GCs proliferation | Mouse | G. [Yao *et al.* (2010](#_ENREF_245)) |
| miR-125b | *Bak*, *Bax*, *Bmf*, *p53* | Prevents follicular atresia | Mouse | [Sen *et al.* (2014](#_ENREF_177)) |
| miR-15a | *BCL2*, *CDC25A* | Growth and maturation of oocytes | Human | Y.W. [Xu *et al.* (2011](#_ENREF_232)) |
| miR-503 | *Gdf9*, *Fshr*, *Esr2*, *ActRIIa*, *Ccnd2*, *ActRIIb* | Expressed during the follicular stage, and becomes undetectable in luteal stage | Mouse | [Gonzalez & Behringer (2009](#_ENREF_65)) |
| miR-449a, miR-449c, miR-222, miR-409a, miR-383, miR-184, let-7 families, miR-10b, miR-26a, miR-99b, miR-27b |  | Play important regulatory roles during recruitment, selection, and dominance of follicles | Cattle | [Salilew-Wondim *et al.* (2014](#_ENREF_173)) |
| miR-181a | *Acvr2a* | Inhibits development of the ovarian follicle | Mouse | Q. [Zhang *et al.* (2013](#_ENREF_257)) |
| miR-497, miR-484, miR-2404, miR-195, miR-16b, miR-128, miR-127, miR-1249, miR-452, miR-27a-3p, miR-2487, miR-193b, miR-185, miR-181a, miR-181b, miR-181c and miR-181d |  | Differentially expressed between DF and SF, and are involved in GnRH signalling | Cattle | [Salilew-Wondim *et al.* (2014](#_ENREF_173)) |
| let-7, miR-10b, miR-26a, miR-99b and miR-27b and bta-miR-92a |  | Most abundant miRNAs in both DF and SF | Cattle | [Salilew-Wondim *et al.* (2014](#_ENREF_173)) |
| miR-132, miR-212, miR-182, miR-96, miR-335, miR-708, miR-221, miR-21-3p, miR-335, miR-214 |  | Differentially expressed between bovine DF and SF | Cattle | [Gebremedhn *et al.* (2015](#_ENREF_61)) |
| miR-125a, miR-127, miR-145, miR-208 |  | Differentially expressed among different stage oocytes | Cattle | [Tesfaye *et al.* (2009](#_ENREF_199)) |
| miR-30, miR-16, let-7 |  | Highly expressed in GV oocytes | Mouse | [Murchison *et al.* (2007](#_ENREF_144)) |
| miR-424, miR-10b |  | Abundant in GV oocytes and might play a role during zygotic genome activation | Cattle | [Tripurani *et al.* (2010](#_ENREF_204)) |
| miR-205 | *PTX3* | Maturation of oocytes | Pig | [Li *et al.* (2016*a*](#_ENREF_102)) |
| miR-133b | *TAGLN2* | Maturation of oocytes | Human, Mouse | [Xiao *et al.* (2014](#_ENREF_228)) |
| miR-27b | *PPARγ* | Fatty acid metabolism during oocyte maturation | Pig | [Song *et al.* (2016](#_ENREF_186)) |
| miR-496, miR-297, miR-292-3P, miR-99a, miR-410, miR-145, miR-515-5p, miR-410, miR-206, miR-423, miR-188, miR-22, miR-34c, miR-181c, miR-467a, miR-190, miR-153, let-7d, miR-122a, miR-182*, miR-140*, miR-365, miR-128a, miR-380-5p, miR-518b, miR-519e, miR-484, miR-128b, miR-223 |  | Expressed abundantly in mature oocytes | Cattle | [Tesfaye *et al.* (2009](#_ENREF_199)) |
| miR-512-5p,  miR-214, miR-200c, miR-298, miR-208, miR-469, miR-375, miR-541, miR-127, miR-381, miR-25, miR-372, miR207, miR-124a, miR-545, miR-433-5p, miR-468, miR-29b, miR-382, miR-130b, miR-378, miR-125a, miR-192, miR-138, miR-519e*, miR-470, miR-98, miR-542-5p, miR-339, miR-547, miR-10a |  | Abundant in immature oocytes | Cattle | [Tesfaye *et al.* (2009](#_ENREF_199)) |
| miR-144, miR-202, miR-451, miR-652, miR-873 |  | Upregulated in the large healthy follicle | Cattle | [Sontakke *et al.* (2014](#_ENREF_190)) |
| miR-200b, miR-429 | *Zeb1* | LH biosynthesis, luteinization, and ovulation | Mouse | [Hasuwa *et al.* (2013](#_ENREF_71)) |
| miR-132, miR-212 |  | Control ovulation and luteinization process | Mouse | [Fiedler *et al.* (2008](#_ENREF_55)) |
| miR-7a2 | *Glg1*, *Bmp4*, *Ptgfrn* | Regulates pituitary gland activity, FSH and LH biosynthesis | Mouse | [Ahmed *et al.* (2017](#_ENREF_3)) |
| miR-335-5p | *Mapk* | Spindle formation and cytoskeleton dynamics | Mouse | [Cui *et al.* (2013](#_ENREF_37)) |
| miR-21 | *PDCD4* | Follicle survival, ovulation, follicle to luteal transition, MII stage commitment | Pig | [Carletti *et al.* (2010](#_ENREF_24)); [Wright *et al.* (2016](#_ENREF_223)) |
| miR-193a-5p, miR-297, miR-625, miR-602 |  | Upregulated in MII oocyte compared to GV oocytes | Human | Y.W. [Xu *et al.* (2011](#_ENREF_232)) |
| miR-888*, miR-212, miR-662, miR-299-5p, miR-339-5p, miR-20a, miR-486-5p, miR-141*, miR-768-5p, miR-376a, miR-15a |  | Downregulated in MII-stage compared to GV oocytes | Human | Y.W. [Xu *et al.* (2011](#_ENREF_232)) |
| miR-199a-3p, miR-145, miR-31 |  | Higher in follicular stage than luteal stage | Sheep | [McBride *et al.* (2012](#_ENREF_133)) |
| miR-503, miR-21, miR-142-3p |  | Higher in luteal stage than follicular stage | Sheep | [McBride *et al.* (2012](#_ENREF_133)) |
| miR-17-5p, let-7b | *Timp1* | Vascularization of CL | Mouse | [Otsuka *et al.* (2008](#_ENREF_155)) |
| miR-378, miR-22-5p, miR-147, miR-877, miR-24, miR-107, miR-148b | *IFNGR1* | Predominant in non-regressed CL | Cattle | [Ma *et al.* (2011](#_ENREF_128)) |
| miR-99a, miR-26a, miR-455, miR-190a, miR-320 and miR-186 |  | Upregulated in regressed CL | Cattle | [Ma *et al.* (2011](#_ENREF_128)) |
| miR-146a | *IRAK1*, *TRAF6* | Regulates follicular atresia | Human | X. [Chen *et al.* (2015](#_ENREF_30)) |
| let-7g | *CCND2*, *BCL-XL* | Upregulated in apoptotic follicles | Pig | [Cao *et al.* (2015](#_ENREF_22)) |
| miR-26b | *ATM* | Enhances follicular atresia | Pig | [Lin *et al.* (2012](#_ENREF_109)) |
| let-7a, let-7b, let-7c, and let-7i |  | Downregulated in atretic follicle | Pig | [Cao *et al.* (2015](#_ENREF_22)) |
| miR-34 family |  | Fertility biomarker for both gametes | Cattle | [Tscherner *et al.* (2014](#_ENREF_205)) |
| miR-29a |  | Follicular development | Cattle | [Hossain *et al.* (2009](#_ENREF_79)) |
| miR-376a | *Grp78* | Regulates follicular to luteal transition | Rat | [Iwamune *et al.* (2014](#_ENREF_86)) |
| miR-136-3p | *Lhr* | Oogenesis | Rat | [Iwamune *et al.* (2014](#_ENREF_86)) |
| miR-378, miR-383, miR-21, miR-17-5p, let-7b |  | Regulates follicular and luteal development |  | [Donadeu *et al.* (2012](#_ENREF_52)) |

**Table S4.** Functions of microRNAs (miRNAs) in the regulation of steroidogenic cells and hormonal balance. GnRH, gonadotropin-releasing hormone; LH, luteinizing hormone; FSH, follicle-stimulating hormone; AR, androgen receptor; UTR, untranslated region; Bt2cAMP, dibutyryl cyclic AMP; MLTC-1, murine Leydig tumor cell line-1; GC, granulosa cell; TC, thecal cell; hCG, human chorionic gonadotropin.

| **miRNA** | **Target genes** | **Function** | **Species** | **Reference** |
| --- | --- | --- | --- | --- |
| miR-155, miR-200 family, and miR-429 family | *Cebpb*, *Zeb1* | Regulates *Pou2f1* and *Meis1* (both are targets for *Zeb1*) and GnRH activity | Mouse | [Messina & Prevot (2017](#_ENREF_138)) |
| miR-200b, miR-429 | *Zeb1* | LH biosynthesis, luteinization, and ovulation | Mouse | [Hasuwa *et al.* (2013](#_ENREF_71)) |
| miR-7a2 | *Glg1*, *Bmp4*, *Ptgfrn* | Regulates pituitary gland functions, FSH and LH biosynthesis | Mouse | [Ahmed *et al.* (2017](#_ENREF_3)) |
| miR-471 | *Foxd1*, *Dsc1* | Metabolism and adhesion of Sertoli cells | Mouse | [Panneerdoss *et al.* (2012](#_ENREF_156)) |
| miR-23b | *Pten*, *Eps15* | Focal adhesion, cytoskeleton regulation | Rat | [Nicholls *et al.* (2011](#_ENREF_146)) |
| miR-133b | *GLI3* | Sertoli cell proliferation | Human | [Yao *et al.* (2016](#_ENREF_244)) |
| miR-762 | *RNF4* | Growth and proliferation of Sertoli cells | Pig | C. [Ma *et al.* (2016](#_ENREF_125)) |
| miR-135b, miR-185, miR-297, miR-299-3p, miR-34a, miR-34c, miR-371-3p, miR-421, miR-449a, miR-449b, miR-634, miR-654-5p, miR-9 |  | Suppress AR expression by directly binding to target sequences in AR 3′-UTR | Human | [Shih *et al.* (2015](#_ENREF_180)) |
| miR-32, miR-148a, miR-99a, miR-21, miR-221 |  | Transcriptionally regulated by AR | Human | [Shih *et al.* (2015](#_ENREF_180)) |
| miR-463, miR-471, miR-201, miR-743A, miR-471-5p, miR-741, miR-463, miR-880, miR-878-5p, miR-871, miR-201, miR -547 |  | Express in androgen-dependent manner in the testes and Sertoli cells | Mouse | [Panneerdoss *et al.* (2012](#_ENREF_156)) |
| miR-23b, miR-30c, miR-30d, miR-17-5p, miR-20, miR-93, miR-106, miR-519, miR-217, miR-329, miR-690 |  | Express in FSH-dependent manner in the testes | Rat | [Nicholls *et al.* (2011](#_ENREF_146)) |
| miR-888, miR-890, miR-891a/b, miR-892a/b |  | Deregulated in the epididymis and seminal vesicle upon vasectomization | Human | [Belleannee *et al.* (2013](#_ENREF_11)*b*) |
| miR-134 | *Cyp11a1* | Steroidogenesis | Rat | [Hu *et al.* (2013](#_ENREF_83)) |
| miR-376b, miR-150, miR-330, miR-138 | *Star* | Steroidogenesis | Rat | [Hu *et al.* (2013](#_ENREF_83)) |
| miR-342 | *Nr5a1* | Steroidogenesis | Rat | [Hu *et al.* (2013](#_ENREF_83)) |
| miR-182, miR-466b | *Ldlr* | Steroidogenesis | Rat | [Hu *et al.* (2013](#_ENREF_83)) |
| miR-183, miR-96, miR-19a | *Abca1* | Steroidogenesis | Rat | [Hu *et al.* (2013](#_ENREF_83)) |
| miR-542 | *Abcg1* | Steroidogenesis | Rat | [Hu *et al.* (2013](#_ENREF_83)) |
| miR-132 | *Srebp-1c* | Steroidogenesis | Rat | [Hu *et al.* (2013](#_ENREF_83)) |
| miR-214 | *Ldlr* | Steroidogenesis | Rat | [Hu *et al.* (2013](#_ENREF_83)) |
| miR-202-5p, miR-202-3p, miR-140-3p, miR-140-5p |  | Differentiation of testes and Leydig cell formation | Mouse | [Eggers *et al.* (2014](#_ENREF_53)) |
| miR-212, miR-183, miR-132, miR-182, miR-96, miR-138, miR-19a |  | Express Bt2cAMP dependently in MLTC-1 Leydig cells | Mouse | [Hu *et al.* (2013](#_ENREF_83)) |
| miR-302a, miR-491-3p, miR-574-5p, miR-297, miR-122, miR-1275, miR-373, miR-185, miR-193b, miR-19b, let-7a |  | Upregulated in azoospermic or idiopathic infertile patients | Human | [Ghorbian (2012](#_ENREF_62)) |
| miR-34c-5p, miR-122, miR-146b-5p, miR-509-5p, miR-29c, miR-34b, miR-520d-3p, miR-383, miR-100, miR-512-3p, miR-16, miR-23b, miR26a-1 |  | Downregulated in azoospermic or idiopathic infertile patients | Human | [Ghorbian (2012](#_ENREF_62)) |
| miR-133b | *TAGLN2* | Growth and maturation of oocytes and GCs | Human, mouse | [Xiao *et al.* (2014](#_ENREF_228)) |
| miR-224 | *Smad4* | Regulates GC proliferation | Mouse | G. [Yao *et al.* (2010](#_ENREF_245)) |
| miR-503, miR-383 |  | Regulates GC proliferation | Mouse | [Lei *et al.* (2010](#_ENREF_100)); [Tripurani *et al.* (2010](#_ENREF_204)); G. [Yao *et al.* (2010](#_ENREF_245)) |
| miR-21 |  | Regulates maintenance of GCs | Mouse | [Carletti *et al.* (2010](#_ENREF_24)) |
| miR-21 |  | Prevents apoptosis of preovulatory GCs | Mouse | [Carletti *et al.* (2010](#_ENREF_24)) |
| miR-132, miR-212 | *Ctbp1* | Regulates differentiation of GCs | Mouse | [Fiedler *et al.* (2008](#_ENREF_55)) |
| miR-18a-5p, miR-582-5p, miR-301b, miR-129-2-3p |  | Regulate ovarian follicle development | Cattle | [Zielak-Steciwko *et al.* (2014](#_ENREF_262)) |
| miR-548ap-5p, miR-548j, miR-539-3p, miR-142-5p, miR-144-5p, miR-126-5p, miR-126-3p, miR-487a, miR-454-5p, miR-223-3p, miR-624-5p, miR-30a-5p, miR-335-5p, miR-889, miR-10b-3p, miR-154-5p, miR-655, miR-4732-5p, miR-32-5p, miR-451a, let-7i-3p, miR-10b-5p, miR-20b-5p, miR-30a-3p, miR-196b-5p, miR-363-3p, miR-429, miR-194-5p, miR-223-5p, miR-379-5p, miR-584-5p, miR-656, miR-106a-5p, miR-144-3p, miR-146a-5p, miR-487b, miR-142-3p, miR-324-5p, miR-377-3p, miR-369-3p, miR-374a-5p, miR-409-5p, miR-548b-5p, miR-1185-2-3p, miR-29b-3p, miR-382-3p, miR-494, miR-98, miR-199b-5p, miR-10a-5p, miR-335-3p, miR-411-5p, let-7f-2-3p, miR-96-5p, miR-483-3p, miR-340-5p, miR-16-5p |  | Abundant in mural GCs | Human | [Velthut-Meikas *et al.* (2013](#_ENREF_213)) |
| miR-129-2-3p, miR-129-5p, miR-1273e, miR-4488, miR-4461, miR-181a-2-3p, miR-1290, miR-34c-3p, miR-196a-5p, miR-4792, miR-874, miR-3651, miR-135a-5p, miR-873-3p, miR-1291, miR-876-5p, miR-181a-3p, miR-1275, miR-4497, miR-181c-3p, miR-320c, miR-23b-5p, miR-378g, let-7c, miR-320d, miR-1292, miR-125b-5p, miR-181b-5p, miR-320b, miR-202-3p, miR-1180, miR-4485, miR-181a-5p |  | Abundant in cumulus GCs | Human | [Velthut-Meikas *et al.* (2013](#_ENREF_213)) |
| miR-21-5p, miR-21-3p, miR-150, miR-409a, miR-142-5p, miR-378, miR-222, miR-155, miR-199a-5p | *HIF1A*, *VEGFA*, *ETS1*, *MSH2* | Promotes follicular atresia | Cattle | [Donadeu *et al.* (2017](#_ENREF_50)) |
| miR-199-3p, miR-125b, miR-145, miR-31, miR-503, miR-21, miR-142-3p |  | Detected in both GCs and TCs | Sheep | [McBride *et al.* (2012](#_ENREF_133)) |
| miR-26b | *ATM* | Promotes follicular atresia and apoptosis of GCs | Pig | [Lin *et al.* (2012](#_ENREF_109)) |
| Let-7g | *CCND2*, *BCL-XL* | Promotes follicular atresia | Pig | [Cao *et al.* (2015](#_ENREF_22)) |
| miR-17, miR-211, miR-542 | *StAR*, *IL-lb*, *COX-2* | Regulates steroidogenesis in follicular cells | Human | [Toloubeydokhti *et al.* (2008](#_ENREF_200)) |
| miR-34a | *INHBB* | Regulates GC apoptosis | Pig | [Tu *et al.* (2014](#_ENREF_206)) |
| miR-26b | *Smad4* | Regulates GC proliferation | Pig | [Liu *et al.* (2014](#_ENREF_112)) |
| miR-26b | *SMAD4*, *BCL2* | Promotes apoptosis of GCs | Pig | [Liu *et al.* (2014](#_ENREF_112)) |
| miR-423-5p,  miR-378 | *CYP19A1* | Regulates synthesis of oestradiol GCs | Pig | [Sui *et al.* (2014](#_ENREF_194)); S. [Xu *et al.* (2011](#_ENREF_231)) |
| miR-378 | *CYP19A1* | Regulates oestradiol production in ovary | Pig | S. [Xu *et al.* (2011](#_ENREF_231)) |
| miR-375 | *SP-1* | Reduces E2 synthesis | Pig | [Yu *et al.* (2016](#_ENREF_249)) |
| miR-132 | *Nurr1* | Oestradiol biosynthesis in GCs | Mouse | [Wu *et al.* (2015](#_ENREF_226)) |
| miR-133b | *FOXL2* | Oestradiol production in GCs | Human, mouse | [Menon *et al.* (2013](#_ENREF_137)) |
| miR-122 |  | Regulates *Lrbp* expression in ovaries | Rat | [Menon *et al.* (2013](#_ENREF_137)) |
| miR-132, miR-212, miR-21, miR-122 |  | Expressed in a LH/hCG-dependent manner in preovulatory follicles | Mouse, rat | [Carletti *et al.* (2010](#_ENREF_24)); [Fiedler *et al.* (2008](#_ENREF_55)); [Menon *et al.* (2013](#_ENREF_137)) |
| miR-212, miR-183, miR-182, miR-132, miR-370, miR-377, miR-96 |  | Expressed in an oestradiol (17α-E2)-dependent manner in adrenal cells | Rat | [Hu *et al.* (2013](#_ENREF_83)) |
| miR-383 | *Rbms1* | Oestradiol biosynthesis in GCs | Mouse | [Yin *et al.* (2012](#_ENREF_247)) |
| miR-23b, miR-29a, miR-30d |  | Express in GCs in a FSH-dependent manner | Rat | N. [Yao *et al.* (2010](#_ENREF_246)) |
| miR-16, miR-24, miR-25, miR-122, miR-145, miR-182, miR-18, miR-125a, miR-147, miR-32, miR-103, miR-143, miR-150, miR-152, miR-153, miR-191 |  | Increases progesterone release in GCs | Human | [Sirotkin *et al.* (2009](#_ENREF_182)) |
| let-7b, let-7c, miR-15a, miR-17-3p, miR-96, miR-92, miR-108, miR-133b, miR-134, miR-135, miR-146, miR-181a, miR-1, miR-19a, miR-20, miR-27a, miR-28, miR-29a, miR-98, miR-125b, miR-126, miR-137, miR-183, miR-184, miR-31, miR-101, miR-105, miR-107, miR-128, miR-129, miR-132, miR-140, miR-141, miR-142, miR-151, miR-188 |  | Inhibits progesterone release in GCs | Human | [Sirotkin *et al.* (2009](#_ENREF_182)) |
| let-7a, let-7b, let-7c, mir-16, mir-17-3p, mir-24, mir-25, mir-26a, mir-108, mir-122, mir-124, mir-133B, mir-134, mir-135, mir-145, mir-146, mir-155, mir-182, let-7d, let-7g, mir-18, mir-19a, mir-20, mir-27a, mir-28, mir-29a, mir-98, mir-100, mir-125a, mir-125b, mir-126, mir-136, mir-137, mir-139, mir-147, mir-148, mir-149, mir-183, mir-184, mir-7, mir-9, mir-10a, mir-21, mir-22, mir-23a, mir-23b, mir-30a-3p, mir-31, mir-32, mir-34a, mir-105, mir-128, mir-129, mir-132, mir-133A, mir-140, mir-141, and mir-188 |  | Inhibits testosterone biosynthesis in GCs | Human | [Sirotkin *et al.* (2009](#_ENREF_182)) |
| mir-107 |  | Increases testosterone biosynthesis in GCs | Human | [Sirotkin *et al.* (2009](#_ENREF_182)) |
| mir-15a, mir-24, mir-25, mir-26a, mir-95, mir-96, mir-92, mir-108, mir-122, mir-124, mir-135, mir-144, mir-146, let-7d, let-7g, mir-1, mir-18, mir-19a, mir-20, mir-27a, mir-28, mir-29a, mir-98, mir-125a, mir-125b, mir-126, mir-137, mir-139, mir-148, mir-149, mir-184, mir-7, mir-10a, mir-22, mir-30a-3p, mir-31, mir-32, mir-34a, mir-101, mir-103, mir-105, mir-128, mir-129, mir-132, mir-133A, mir-140, mir-150, mir-151, mir-152, mir-187, and mir-188 |  | Inhibits oestradiol release in GCs | Human | [Sirotkin *et al.* (2009](#_ENREF_182)) |
| miR-320 | *E2f1*,  *Sf-1* | Regulates the functions of GCs | Mouse | [Yin *et al.* (2014](#_ENREF_248)) |
| miR-497, miR-15b |  | Expressed in ovaries in a testosterone-dependent manner | Sheep | [Luense *et al.* (2011](#_ENREF_119)) |
| miR-145 | *Acvr1b* | Suppresses proliferation of GCs | Mouse | [Yan *et al.* (2012](#_ENREF_234)) |
| miR-23a | *XIAP* | Pro-apoptotic role on GCs | Human | [Yang *et al.* (2012](#_ENREF_243)) |
| miR-23a, miR-27a | *SMAD5* | Enhances the apoptosis of GCs | Human | [Nie *et al.* (2015](#_ENREF_147)) |
| Let-7g | *TGFBR1* | Promotes follicular atresia | Pig | [Zhou *et al.* (2015](#_ENREF_261)) |
| miR-15a, miR-96, miR-92, miR-124, miR-18, miR-29a, miR-125a, miR-136, miR-147, miR-183, miR-32 | *BAX* | Promotes apoptosis of GCs | Human | [Sirotkin *et al.* (2010](#_ENREF_181)) |

**Table S5**. Functions of microRNAs (miRNAs) in fertilization, implantation, and germ-layer specification. IVF, *in vitro* fertilization; CL, corpus luteum; ESC, embryonic stem cell.

| **miRNA** | **Target genes** | **Function** | **Species** | **Reference** |
| --- | --- | --- | --- | --- |
| miR-532-5p, miR-374b-5p, miR-564 |  | Fertility biomarkers of spermatozoa | Human | [Salas-Huetos *et al.* (2014](#_ENREF_172)) |
| miR-34c |  | Plays role in first zygotic cell division | Mouse | [Liu *et al.* (2012](#_ENREF_115)) |
| let-7 family, miR-30 family, miR-16 |  | Maternal miRNAs | Mouse | [Svoboda & Flemr (2010](#_ENREF_195)) |
| miR-17-92 cluster, miR103,  miR-342, miR-200 family |  | Maternal-zygotic miRNAs | Mouse | [Svoboda & Flemr (2010](#_ENREF_195)) |
| miR-290 cluster |  | Zygotic miRNAs | Mouse | [Svoboda & Flemr (2010](#_ENREF_195)) |
| miR-21-5p |  | High in poor IVF responders | Human | [Karakaya *et al.* (2015](#_ENREF_91)) |
| miR-21-3p |  | Low in poor IVF responders | Human | [Karakaya *et al.* (2015](#_ENREF_91)) |
| miR-34b/c, miR-449a/b/c |  | Sperm-borne miRNAs | Mouse | [Yuan *et al.* (2015](#_ENREF_253)) |
| miR-191, miR-16, miR-146a |  | Sperm-borne miRNAs | Mouse | [Yang *et al.* (2016](#_ENREF_238)) |
| let-7a, miR-16, miR-21, miR-31, miR-101, miR-145, miR-182, miR-192, miR-194, miR-210, miR-212 |  | Expressed in both oocytes and blastocysts | Human | [Tulay *et al.* (2015](#_ENREF_207)) |
| miR-7-2, miR-15a, miR-23, miR-34, miR-128, miR-130, miR-155 |  | Expressed specifically in blastocysts | Human | [Tulay *et al.* (2015](#_ENREF_207)) |
| miR-205, miR-150, miR-122, miR-96, miR-146a, miR-146b-5p |  | Highly expressed in immature oocytes | Cattle | [Abd El Naby *et al.* (2013](#_ENREF_1)) |
| miR-125a, miR-25, miR-127, miR-145, miR-208, miR-496 |  | Expressed differentially in different stages of early embryonic development | Cattle | [Tesfaye *et al.* (2009](#_ENREF_199)) |
| Let-7 family | *Muc1* | Regulates implantation of the embryo | Mouse | [Inyawilert *et al.* (2015](#_ENREF_85)) |
| miR-30 family |  | Plays role in endometrium during implantation | Human | [Moreno-Moya *et al.* (2014](#_ENREF_142)) |
| miR-17-92 cluster | *E2Fs*, *TGFβ* | Role on decidualization (morphological and functional changes of the endometrium in preparation for pregnancy) and implantation | Human, Mice | [Mogilyansky & Rigoutsos (2013](#_ENREF_141)) |
| miR-21 | *Reck* | Regulates implantation of embryos | Mouse | [Carletti & Christenson (2009](#_ENREF_23)) |
| miR-21 |  | Marker of a live embryo | Mouse | [Hu *et al.* (2008](#_ENREF_82)); [Luense *et al.* (2009](#_ENREF_118)) |
| miR-29a |  | Highly expressed during implantation | Rat | [Xia *et al.* (2014](#_ENREF_227)) |
| miR-101, miR-199* | *Cox-2* (*Ptgs2*) | Regulates implantation of embryos | Mouse | [Chakrabarty *et al.* (2007](#_ENREF_25)) |
| miR-705 | *Mmp9* | Plays role in implantation of embryos | Mouse | [Carletti & Christenson (2009](#_ENREF_23)) |
| miR-125b, miR-145, mir-31, miR-503, miR-21 |  | Vasculature and angiogenesis during the follicular and luteal cycle. | Cattle | [Donadeu *et al.* (2012](#_ENREF_52)) |
| miR-378 | *IFNGR1*, *CYP19A1* | Plays role in the maintenance of CL | Cattle | [Donadeu *et al.* (2012](#_ENREF_52)); [Ma *et al.* (2011](#_ENREF_128)) |
| miR-17-5p, let-7 | *Timp1* | Regulates angiogenesis in CL | Mouse | [Otsuka *et al.* (2008](#_ENREF_155)) |
| miR-199a-3p, miR-145, miR-503 |  | Differentially expressed between CL and corpus albicans | Sheep | [Donadeu *et al.* (2012](#_ENREF_52)) |
| miR-200 | *Zeb1*, *Zeb2* | Plays anti-implantation role | Mouse | [Jimenez *et al.* (2016](#_ENREF_87)) |
| miR-200a | *Pten* | Plays anti-implantation role | Mouse | [Shen *et al.* (2013](#_ENREF_179)) |
| miR-22 | *Tiam1*, *Rac1* | Plays anti-implantation role | Mouse | [Ma *et al.* (2015](#_ENREF_127)) |
| miR-145 | *Igf1*, *Igf1r* | Blocks embryo-epithelial juxtacrine communication | Mouse | [Kang *et al.* (2015](#_ENREF_90)) |
| miR-451, miR-424, miR-125b, miR-30b |  | Downregulated at high levels of progesterone during implantation | Human | [Li *et al.* (2011](#_ENREF_105)) |
| miR-378a | *NODAL* | Regulates trophoblast cell proliferation | Human | [Luo *et al.* (2012](#_ENREF_120)) |
| miR-376c | *NODAL*, *TGFβ* | Regulates trophoblast cell proliferation | Human | [Fu *et al.* (2013](#_ENREF_56)) |
| miR-195 | *ActRIIA* | Regulates trophoblast migration | Human | [Bai *et al.* (2012](#_ENREF_6)) |
| miR-182 |  | Inhibits apoptosis of trophoblasts | Human | [Pineles *et al.* (2007](#_ENREF_158)) |
| miR-155 | *CCND1* | Inhibits proliferation of trophoblasts | Human | [Dai *et al.* (2012](#_ENREF_43)) |
| miR-675 | *NOMO1* | Inhibits proliferation of trophoblasts | Human | [Gao *et al.* (2012](#_ENREF_59)) |
| miR-29b | *MCL1*, *MMP2*, *VEGFA*, *ITGB1* | Induces apoptosis of trophoblasts | Human | [Li *et al.* (2013](#_ENREF_104)) |
| miR-210 |  | Inhibits migration of trophoblasts | Human | [Zhang *et al.* (2012](#_ENREF_258)) |
| miR-34a |  | Inhibits migration of trophoblasts | Human | [Umemura *et al.* (2013](#_ENREF_211)) |
| miR-16 | *CCNE1*, *VEGFA* | Suppresses vasculature and angiogenesis during implantation of embryos | Human | [Wang *et al.* (2012](#_ENREF_218)) |
| miR-302 cluster, miR-371, miR-372, miR-373, miR-520 cluster, miR-17/92 cluster, miR-106a-92 |  | Expressed in ESCs | Human | [Berardi *et al.* (2012](#_ENREF_12)) |
| miR-106a, miR-93, miR-20, miR-17-5p, miR-290 |  | Expressed in ESCs | Mouse | [Berardi *et al.* (2012](#_ENREF_12)) |
| miR-124a |  | Expressed in gastrulation-stage embryos | Human | [Berardi *et al.* (2012](#_ENREF_12)) |
| miR-200a, miR-200b, miR-200c, miR-141, miR-429 |  | Expressed in gastrulation-stage embryos | Mouse | [Berardi *et al.* (2012](#_ENREF_12)) |
| miR-9, miR-124a, miR-155, miR-708 |  | Promotes differentiation of ESCs | Mouse | [Marson *et al.* (2008](#_ENREF_132)) |
| miR-24-3p, miR-24-2-5p | *Oct4*, *Nanog*, *Klf4*,  *c-Myc* | Promotes differentiation of ESCs | Mouse | [Lee *et al.* (2016](#_ENREF_99)) |
| miR-421 | *Oct4*, *Smad5*, *Id2* | Regulates *BMP* signalling | Mouse | [Hadjimichael *et al.* (2016](#_ENREF_69)) |
| miR-303~367 | *NODAL* | Patterning and specification of embryonic germ layers | Human | [Vidigal & Ventura (2012](#_ENREF_236)) |
| miR-290~295 | *Nodal* | Patterning and specification of embryonic germ layers | Mouse | [Vidigal & Ventura (2012](#_ENREF_236)) |
| miR-21 |  | Facilitates commitment of mesodermal tissues | Mouse, Human | [Eguchi *et al.* (2013](#_ENREF_54)); [Kang *et al.* (2013](#_ENREF_89)) |
| miR-127 | *Lefty2* | Promotes ESCs to mesendoderm lineage | Mouse | H. [Ma *et al.* (2016](#_ENREF_126)) |
| let-7 | *Acvr1b* | Regulates germ layer specification in ESCs | Mouse | [Colas *et al.* (2012](#_ENREF_34)) |
| miR-18 | *Smad2* | Regulates germ layer specification in ESCs | Mouse | [Colas *et al.* (2012](#_ENREF_34)) |
| miR-200 family (miR-200c, miR-141, miR-200b, miR-200a, miR-429) | *Snail* | Facilitates mesoderm commitment in ESCs | Mouse | [Gill *et al.* (2011](#_ENREF_63)) |
| miR-145 | *OCT4*, *SOX2*, *KLF4* | Promotes mesoderm and ectoderm commitment in ESCs | Human | [Xu *et al.* (2009](#_ENREF_230)) |
| miR-290, miR-93, miR-17-5p |  | Facilitates commitment of mesoderm | Mouse | [Berardi *et al.* (2012](#_ENREF_12)) |
| miR-145, miR-302 |  | Facilitates commitment of mesoderm | Human | [Berardi *et al.* (2012](#_ENREF_12)) |
| miR-23a |  | Suppresses endoderm and ectoderm lineage commitment in ESCs | Mouse | [Hadjimichael *et al.* (2016](#_ENREF_69)) |
| miR-191, miR-16-1 | *Smad2* | Inhibits mesoderm formation in ESCs | Mouse | [Hadjimichael *et al.* (2016](#_ENREF_69)) |
| miR-145, miR-10a, miR-24, miR-375, miR-122, miR-192, miR-196a, miR-196b |  | Facilitates endoderm commitment | Human | [Berardi *et al.* (2012](#_ENREF_12)) |
| miR-93, miR-338-5p, miR-340-3p |  | Facilitates endoderm commitment | Mouse | [Berardi *et al.* (2012](#_ENREF_12)) |
| miR-338-5p, miR-340-3p | *Hdac* | Promotes endodermal differentiation of ESCs | Mouse | [Fu *et al.* (2011](#_ENREF_57)) |
| miR-375 | *TIMM8A* | Promotes endoderm commitment of ESCs | Human | [Hinton *et al.* (2010](#_ENREF_77)) |
| miR-125, miR-30b, miR-30c |  | Promotes ectoderm commitment | Human | [Berardi *et al.* (2012](#_ENREF_12)) |
| miR-29c, miR-125a, miR-376a, miR-297, miR-96, miR-21, let-7, miR-424 and miR-214 |  | Promotes ectoderm commitment | Mouse | [Berardi *et al.* (2012](#_ENREF_12)) |
| miR-30b | *EED* | Regulates embryonic ectoderm development | Human | [Song *et al.* (2011](#_ENREF_187)) |
| miR-125 | *SMAD4* | Neural lineage commitment in ESCs | Human | [Boissart *et al.* (2012](#_ENREF_19)) |
| miR-146b-5p | *SMAD4* | Neural lineage commitment in ESCs | Human | [Zhang *et al.* (2017](#_ENREF_256)) |
| miR-17-5p | *Rbl2* | Lung epithelial cell proliferation and differentiation | Mouse | [Lu *et al.* (2007](#_ENREF_117)) |
| miR-125b-2 | *Lin28*, *Dies1* | Represses ectoderm commitment in ESCs | Mouse | [Deng *et al.* (2015](#_ENREF_47)) |
| miR-125, miR-30b, miR-30c |  | Specific to trophectoderm | Human | [Berardi *et al.* (2012](#_ENREF_12)) |
| miR-297, miR-214, miR-96, miR-125a, miR-21, miR-93, miR-424, miR-29c, let-7, miR-376a |  | Specific to trophectoderm | Mouse | [Berardi *et al.* (2012](#_ENREF_12)) |
| miR-297, miR-96, miR-214, miR-125a, miR-424, miR-21, miR-29c, let-7 |  | Upregulated during trophectoderm formation | Mouse | [Viswanathan *et al.* (2009](#_ENREF_216)) |
| miR-376a |  | Downregulated during trophectoderm formation | Mouse | [Viswanathan *et al.* (2009](#_ENREF_216)) |
| miR-101, miR-125a, miR-127, miR-133, miR-139, miR-140, miR-143, miR-145, miR-193, miR-199, miR-27, miR-29, miR-411, miR-497 |  | Expressed in endometrium | Human | [Hiroki *et al.* (2010](#_ENREF_78)) |
| miR-10a, miR-27a, miR-29c, miR-323, miR-331-5p, miR-339-3p, miR-374-5p, and miR-935 |  | Associated with the spontaneous foetal arrest | Pig | [Wessels *et al.* (2013](#_ENREF_221)) |
| miR-296-5P, miR-150, miR-17P-5P, miR-18a, and miR-19 |  | Associated with angiogenesis of endometrial tissues and differentially expressed between healthy and arresting conceptus-attachment site | Pig | [Bidarimath *et al.* (2015](#_ENREF_14)) |
| miR-20b, miR-17-5P, miR-18a, miR-15b-5P, and miR-222 |  | Differentially expressed between endometrium and trophoblasts | Pig | [Bidarimath *et al.* (2015](#_ENREF_14)) |
| miR-126-5P, miR-296-5P, miR-16, and miR-17-5P |  | Angiogenic miRNAs participate in the conceptus-endometrial crosstalk | Pig | [Bidarimath *et al.* (2017](#_ENREF_15)) |
